# Computationally designed mRNA-launched protein nanoparticle immunogens elicit protective antibody and T cell responses in mice

**DOI:** 10.1126/scitranslmed.adu2085

**Published:** 2025-10-15

**Authors:** Grace G. Hendricks, Lilit Grigoryan, Mary Jane Navarro, Nicholas J. Catanzaro, Miranda L. Hubbard, John M. Powers, Melissa Mattocks, Catherine Treichel, Alexandra C. Walls, Jimin Lee, Daniel Ellis, Jing Yang (John) Wang, Suna Cheng, Marcos C. Miranda, Adian Valdez, Cara W. Chao, Sidney Chan, Christine Men, Max R. Johnson, Samantha K. Zepeda, Sebastian Ols, Harold Hui, Sheng-Yang Wu, Victor Lujan, Hiromi Muramatsu, Paulo J.C. Lin, Molly M.H. Sung, Ying K. Tam, Elizabeth M. Leaf, Norbert Pardi, Ralph S. Baric, Bali Pulendran, David Veesler, Alexandra Schäfer, Neil P. King

**Affiliations:** 1 Institute for Protein Design, University of Washington, Seattle, WA 98195, USA; 2 Department of Biochemistry, University of Washington, Seattle, WA 98195, USA; 3 Institute for Immunity, Transplantation and Infection, Stanford University School of Medicine, Stanford University, Stanford, CA 94305, USA; 4 Department of Epidemiology, University of North Carolina at Chapel Hill, Chapel Hill, NC 27599, USA; 5 Department of Microbiology and Immunology, University of North Carolina at Chapel Hill, Chapel Hill, NC 27599, USA; 6 Howard Hughes Medical Institute, Seattle, WA 98195, USA; 7 Graduate Program in Molecular and Cellular Biology, University of Washington, Seattle, WA 98195, USA; 8 Department of Microbiology, Perelman School of Medicine, University of Pennsylvania, Philadelphia, PA 19104, USA; 9 Acuitas Therapeutics, Vancouver, BC V6T 1Z3, Canada; 10 Lead contact

## Abstract

mRNA vaccines and computationally designed protein nanoparticle vaccines were both clinically de-risked and licensed for the first time during the COVID-19 pandemic. These vaccine modalities have complementary immunological benefits that provide strong motivation for their combination. Here we demonstrate proof of concept for genetic delivery of computationally designed protein nanoparticle immunogens. Using SARS-CoV-2 as a model system, we genetically fused a stabilized variant of the Wuhan-Hu-1 spike protein receptor binding domain (RBD) to a protein nanoparticle we previously designed for optimal secretion from human cells. Upon secretion, the nanoparticle formed monodisperse and antigenically intact assemblies displaying 60 copies of the RBD in an immunogenic array. Compared with mRNA vaccines encoding membrane-anchored spike protein and a secreted RBD trimer, an mRNA vaccine encoding the RBD nanoparticle elicited 5- to 28-fold higher titers of neutralizing antibodies in mice. Additionally, the “mRNA-launched” RBD nanoparticle vaccine induced higher frequencies of antigen-specific CD8 T cells than the same immunogen delivered as adjuvanted protein and protected mice from either Wuhan-Hu-1 or Omicron BA.5 challenge. These results establish that delivering computationally designed protein nanoparticle immunogens through mRNA can combine the benefits of both vaccine modalities. More broadly, our data highlight the utility of computational protein design in genetic vaccination strategies.

## One Sentence Summary

Genetic delivery of designed protein nanoparticles displaying the SARS-CoV-2 RBD elicits potent neutralizing antibody and CD8 T cell responses.

## Introduction

The coronavirus disease 2019 (COVID-19) pandemic established that mRNA vaccines can be safe, effective, and rapidly scalable (1–4). This success was made possible by decades of research focused on optimizing both the mRNA backbone (i.e., nucleoside modifications (5, 6), 5′ capping strategies (7, 8), and structured untranslated regions (UTRs) (9, 10)) and the delivery vehicle (i.e., lipid nanoparticle (LNP) formulations (11, 12)). Numerous studies of mRNA-LNP vaccines have established several advantages of this modality, including induction of antigen-specific CD8 T cell responses (13, 14) and intrinsic adjuvant activity deriving from the LNP (15, 16). However, there is a key aspect of mRNA vaccine development that has been understudied: optimizing the immunogenicity of the encoded protein.

Multivalent antigen display on self-assembling protein nanoparticles has become a widespread and successful approach for improving the immunogenicity of protein subunit vaccines (17). Until recently, most studies in this area have relied on naturally occurring protein nanoparticles such as ferritin, lumazine synthase, or virus-like particles (VLPs) (18, 19). However, computationally designed protein nanoparticles have emerged as a robust and versatile platform for multivalent antigen display that provides control over many aspects of immunogen structure, including antigen copy number and display density (20, 21). Antigen display at high density drives clustering or cross-linking of antigen-specific B cell receptors (BCRs), leading to both improved avidity and more potent BCR signaling and B cell activation (22). Compared with soluble antigen, particulate immunogens also exhibit superior interactions with the innate immune system, as well as better trafficking to, and retention within, B cell follicles in lymph nodes (23, 24). These mechanisms combine to drive potent antibody responses to protein nanoparticle immunogens. However, despite their immunological advantages and genetic basis, the delivery of protein nanoparticle immunogens as mRNA vaccines has only recently begun to be explored (25–28).

Integrating the complementary strengths of protein nanoparticle immunogens and mRNA vaccines in a single platform could be a powerful approach to vaccine design. Additionally, the rapid and sequence-invariant manufacturing of mRNA could make “mRNA-launched” nanoparticle vaccines a promising platform for pandemic preparedness or pandemic response vaccine development (29). However, to successfully develop this technology, the protein nanoparticle immunogens must be designed such that they are not only produced and assembled within eukaryotic host cells, but also efficiently secreted. This can require careful consideration of the interactions of the encoded amino acid sequence with cellular machinery. For example, we recently discovered that highly hydrophobic segments in computationally designed protein nanoparticles can be interpreted as transmembrane domains during translocation into the endoplasmic reticulum (ER), causing the nanoparticle subunits to partition into the ER membrane and precluding their secretion (30). By developing a computational protocol to identify polar mutations that eliminate such cryptic transmembrane domains without perturbing protein stability and assembly, we improved the secretion of a computationally designed 60-subunit icosahedral nanoparticle by 64-fold (30). This nanoparticle, called I3–01NS, represents a promising scaffold for the development of mRNA-launched nanoparticle immunogens. Here we use severe acute respiratory syndrome coronavirus 2 (SARS-CoV-2) as a model system to evaluate the secretion, antigenicity, immunogenicity, and protective capacity of computationally designed mRNA-launched nanoparticle vaccines. We directly compared the mRNA-launched receptor binding domain (RBD) nanoparticles to two mimics of best-in-class, clinically approved COVID-19 vaccines: an mRNA vaccine encoding prefusion-stabilized, membrane-anchored spike protein and an adjuvanted protein RBD nanoparticle.

## Results

### Immunogen design and characterization

**Figure 1.**
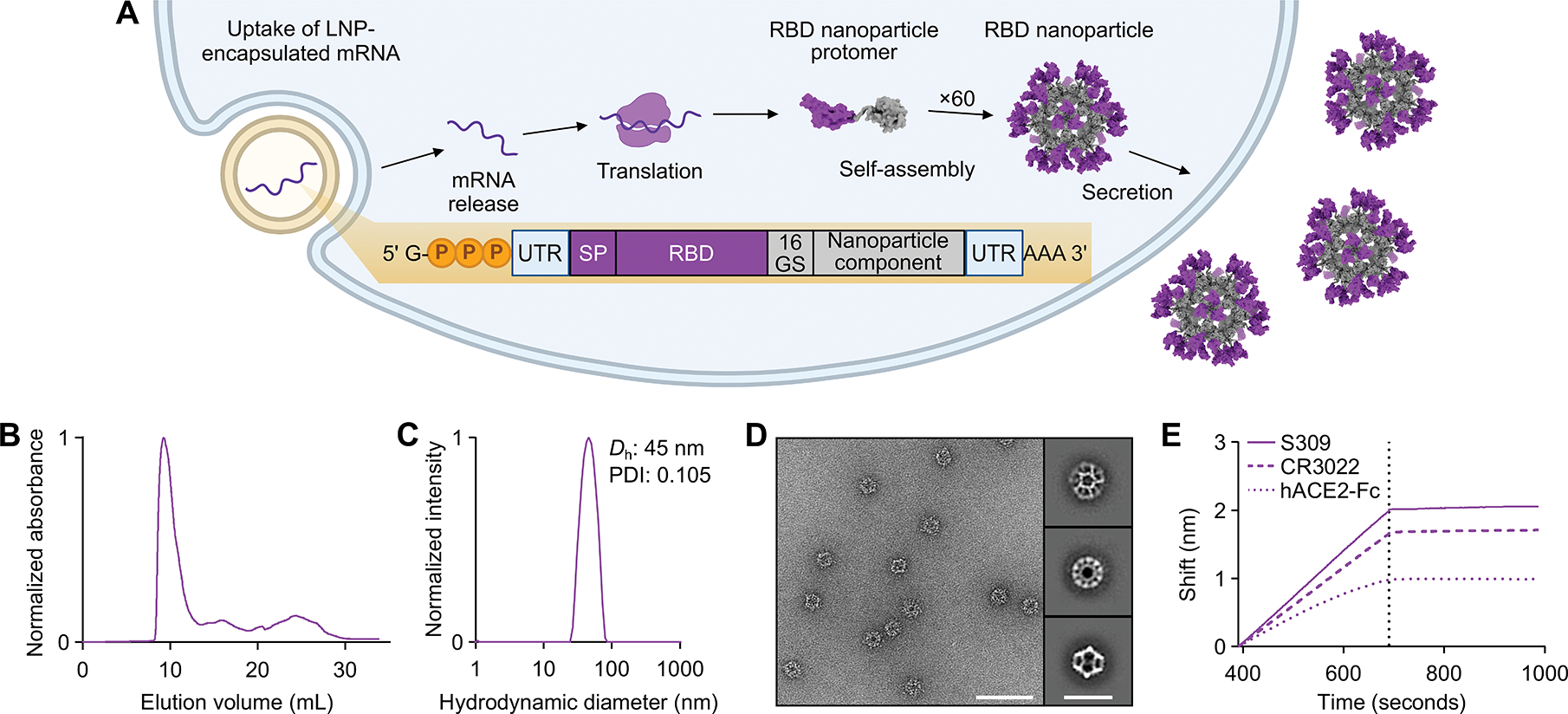
Design and characterization of a secretable SARS-CoV-2 RBD nanoparticle. (A) Schematic of the biogenesis of secreted RBD nanoparticles using mRNA-LNP as an example for method of delivery. The secretory pathway has been omitted for simplicity. UTR, untranslated region; SP, signal peptide; 16 GS, 16-residue glycine/serine linker. The protein models and schematic were rendered using ChimeraX (82) and BioRender.com, respectively. (B) Size exclusion chromatogram of Rpk9-I3–01NS purification. (C) Dynamic light scattering of SEC-purified Rpk9-I3–01NS. *D*_h_, hydrodynamic diameter; PDI, polydispersity index. (D) Representative electron micrograph (scale bar = 50 nm) of negatively stained SEC-purified Rpk9-I3–01NS and 2D class averages (scale bar = 33 nm). (E) Binding of immobilized hACE2-Fc, CR3022, and S309 to SEC-purified Rpk9-I3–01NS as assessed by biolayer interferometry. The dotted vertical line separates the association and dissociation steps.

To generate a secretable RBD nanoparticle, we multivalently displayed the Wuhan-Hu-1 SARS-CoV-2 spike RBD on the exterior surface of the self-assembling protein nanoparticle I3–01NS (30, 31). I3–01NS is a one-component, 60-subunit complex with icosahedral symmetry derived from a naturally occurring bacterial aldolase (32) that we computationally designed for optimal secretion from mammalian cells. We genetically fused the RBD (residues 328–531) to the N terminus of I3–01NS using a 16-residue glycine/serine linker to enable flexible presentation of the antigen extending from the nanoparticle surface (Fig. 1A, and table S1). To mimic the process of expression and secretion during genetic vaccination, we transfected human (Expi293F) cells with codon-optimized plasmid DNA encoding the resultant fusion construct, RBD-I3–01NS. SDS-PAGE of the cell culture supernatant revealed that RBD-I3–01NS did not secrete (fig. S1A). To recover secretion, we swapped out the wild-type Wuhan-Hu-1 SARS-CoV-2 RBD for a stabilized and higher-yielding version, Rpk9, which contains three mutations (Y365F, F392W, V395I) that repack the linoleic acid binding pocket (33, 34). The new fusion construct, Rpk9-I3–01NS, did secrete from cells and was carried forward for purification (table S1 and fig. S1A). Size exclusion chromatography (SEC) of Rpk9-I3–01NS revealed a predominant peak corresponding to the target icosahedral assembly (Fig. 1B), and dynamic light scattering (DLS) and negative stain electron microscopy (nsEM) confirmed a homogenous and monodisperse population of nanoparticles (Fig. 1, C and D). Biolayer interferometry (BLI) with Fc-tagged human angiotensin converting enzyme 2 (hACE2-Fc), the class 4 RBD-directed monoclonal antibody (mAb) CR3022 (35), and the class 3 RBD-directed mAb S309 (36) confirmed that multiple epitopes of Rpk9 were intact and accessible in the context of the I3–01NS nanoparticle (Fig. 1E). To rigorously assess the yield of secreted Rpk9-I3–01NS, we used the purified protein to create a standard curve for supernatant enzyme-linked immunosorbent assays (ELISAs). Based on three independent transfections, Rpk9-I3–01NS was shown to secrete at 29.9 mg/L whereas the original RBD-I3–01NS construct secreted at concentrations below the lower limit of detection of the assay (fig. S1, B to D). These data indicate that Rpk9-I3–01NS nanoparticles secrete efficiently and are biochemically, antigenically, and structurally intact.

### Rpk9-I3–01NS elicited neutralizing antibody responses

**Figure 2.**
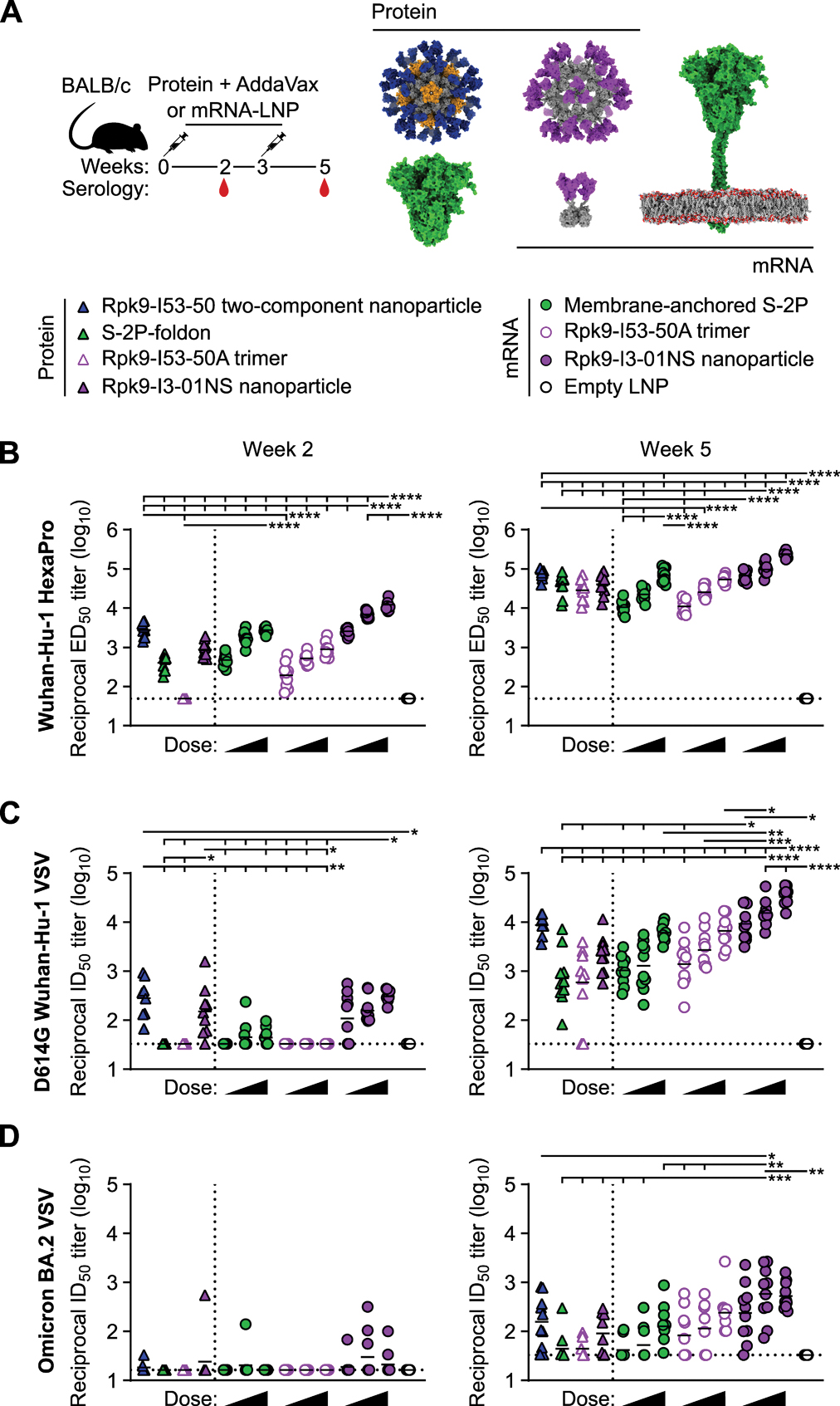
mRNA-launched RBD nanoparticles elicit binding and neutralizing antibody titers in BALB/c mice. **(A)** Study design and groups; n=10 mice/group (except for Empty LNP, where n=5) received either AddaVax-adjuvanted protein (equimolar amounts of RBD: 0.9 μg of RBD per dose for Rpk9-based constructs, 5 μg of spike protein per dose for S-2P-foldon) or nucleoside-modified, LNP-encapsulated mRNA (0.2, 1, or 5 μg per dose). Molecular models of immunogens are not to scale. Rpk9 is shown in blue (Rpk9-I53–50) or purple (Rpk9-I53–50A and Rpk9-I3–01NS). The trimer scaffold shown is in gray (Rpk9-I53–50, Rpk9-I53–50A, and Rpk9-I3–01NS). The pentamer component is shown in orange (Rpk9-I53–50). The S-2P-foldon and membrane-anchored S-2P, shown in green, are adapted from (83). Glycans have been omitted from all models for simplicity. All models were rendered using ChimeraX (82). **(B)** Serum antibody binding titers against Wuhan-Hu-1 SARS-CoV-2 S HexaPro (84) were measured by ELISA. ED_50_, half-maximal effective dilution. **(C)** Serum neutralizing antibody titers against VSV pseudotyped with D614G Wuhan-Hu-1 SARS-CoV-2 spike protein; ID_50_, half-maximal inhibitory dilution. **(D)** Serum neutralizing antibody titers against VSV pseudotyped with Omicron BA.2 SARS-CoV-2 spike protein. In (B to D), each symbol represents an individual animal and the geometric mean titer (GMT) from each group is indicated by a horizontal line. The dotted horizontal line represents the lowest limit of detection for the assay; limits of detection vary between groups. The dotted vertical line separates the protein and mRNA immunized groups. Statistical significance was determined using one-way ANOVA with Tukey’s multiple comparisons test; *p < 0.05; **p < 0.01; ***p < 0.001; ****p < 0.0001. Significant differences where p > 0.0001 have been omitted from (B) for clarity. All significant comparisons are presented for (C) and (D). A full statistical analysis can be found in data file S3.

We assessed the immunogenicity of Rpk9-I3–01NS when delivered as an adjuvanted protein vaccine (“protein-delivered”) and an mRNA vaccine (“mRNA-launched”). To directly evaluate the impact of nanoparticle formation, we also assessed the immunogenicity of protein-delivered and mRNA-delivered “non-assembling” Rpk9-I53–50A trimers, which secreted at 122.4 mg/L in vitro (fig. S1, E to H). I53–50A is a trimeric scaffold that shares 90% amino acid sequence identity with I3–01NS but lacks the hydrophobic interface that drives nanoparticle assembly (37). We also included protein-delivered prefusion-stabilized spike protein ectodomain (S-2P-foldon trimer) as a benchmark immunogen (38–40). Finally, to assess Rpk9-I3–01NS within the current COVID-19 vaccine landscape, we also evaluated the immunogenicity of mRNA-delivered membrane-anchored S-2P trimers (using the exact mRNA sequence of Comirnaty (41, 42)), as well as protein-delivered Rpk9-I53–50 two-component nanoparticles (the Rpk9 version of SKYCovione (33, 43)). Groups of ten BALB/c mice were immunized intramuscularly on weeks 0 and 3 with either AddaVax-adjuvanted protein (equimolar amounts of RBD: 0.9 μg of RBD per dose for Rpk9-based constructs, 5 μg of spike protein per dose for S-2P-foldon) or nucleoside-modified, LNP-encapsulated mRNA (0.2, 1, or 5 μg dose) (Fig. 2A, and table S2 and S3). Additionally, we immunized five mice with lipid nanoparticles not encapsulating any mRNA (“empty” LNPs) as a negative control group. Vaccine-elicited antibody responses were then assessed two weeks post-prime and -boost by serum ELISAs and pseudovirus neutralization assays.

Two weeks post-prime, all RBD nanoparticle vaccines consistently elicited antigen-specific binding and vaccine-matched (D614G Wuhan-Hu-1 VSV) pseudovirus neutralizing antibody titers. By contrast, the non-particulate vaccines consistently elicited antigen-specific binding antibody titers but minimal to no vaccine-matched pseudovirus neutralizing antibody titers (Fig. 2, B and C, fig. S2 and S3). As expected, minimal to no vaccine-mismatched (Omicron BA.2 VSV) pseudovirus neutralizing antibody titers were elicited by any of the vaccines (Fig. 2D and fig. S4). At every dose of mRNA, mRNA-launched Rpk9-I3–01NS elicited ≥4-fold higher antigen-specific binding antibody titers than mRNA-delivered membrane-anchored S-2P (p < 0.0001 for the 5 and 1 μg doses; p < 0.01 for the 0.2 μg dose). Similarly, mRNA-launched Rpk9-I3–01NS elicited >10-fold higher antigen-specific binding antibody titers than secreted Rpk9-I53–50A at every dose of mRNA (p < 0.0001 for the 5 and 1 μg doses, p < 0.001 for the 0.2 μg dose), despite secreting at 4-fold lower concentrations in tissue culture (fig. S1). Further, at the 5 μg dose of mRNA, mRNA-launched Rpk9-I3–01NS elicited ≥7-fold higher vaccine-matched pseudovirus neutralizing antibody titers than both mRNA-delivered membrane-anchored S-2P and secreted Rpk9-I53–50A (p < 0.05 for both comparisons). These observations were consistent with the intrinsically higher immunogenicity of the particulate immunogens when delivered as antigen dose-matched adjuvanted proteins: Rpk9-I3–01NS and Rpk9-I53–50 both elicited ≥5-fold higher vaccine-matched pseudovirus neutralizing antibody titers than both Rpk9-I53–50A (p < 0.05 and p < 0.01, respectively) and S-2P-foldon (p < 0.05 and p < 0.01, respectively).

Following a second immunization, we observed enhanced antigen-specific binding, vaccine-matched and -mismatched pseudovirus neutralizing antibody titers for all vaccines (Fig. 2, B to D, and fig. S2 to S4). Consistent with the post-prime data, mRNA-launched Rpk9-I3–01NS elicited ≥5-fold higher vaccine-matched pseudovirus neutralizing antibody titers at every dose of mRNA compared with mRNA-delivered membrane-anchored S-2P (p < 0.0001 for the 5 and 1 μg doses; p < 0.05 for the 0.2 μg dose) and secreted Rpk9-I53–50A (p < 0.0001 for the 5 μg dose; p < 0.001 for the 1 μg dose; p < 0.05 for the 0.2 μg dose). Additionally, at the 1 μg dose of mRNA, mRNA-launched Rpk9-I3–01NS elicited 11- and 5-fold higher vaccine-mismatched pseudovirus neutralizing antibody titers than mRNA-delivered membrane-anchored S-2P (p < 0.001) and secreted Rpk9-I53–50A (p < 0.01), respectively. When comparing across doses of mRNA, the vaccine-matched and -mismatched pseudovirus neutralizing antibody titers elicited by the 0.2 μg dose of mRNA-launched Rpk9-I3–01NS were comparable to those elicited by the 5 μg dose of mRNA-delivered membrane-anchored S-2P, despite the 25-fold lower dose. When comparing between vaccine modalities, the 5 μg dose of mRNA-launched Rpk9-I3–01NS elicited 4-fold higher vaccine-matched neutralizing antibody titers than the 0.9 μg RBD dose of protein-delivered two-component Rpk9-I53–50 (p < 0.0001). Further, the 1 μg dose of mRNA-launched Rpk9-I3–01NS elicited 4-fold higher vaccine--mismatched pseudovirus neutralizing antibody titers than the 0.9 μg RBD dose of Rpk9-I53–50 (p < 0.05). Finally, as expected, we also detected antibody responses to the I3–01NS, I53–50A, and I53–50 scaffolds (fig. S5).

Several conclusions can be drawn from these data. First, secreted RBD nanoparticles can be effectively delivered as mRNA vaccines and elicit potent humoral responses. Second, as shown previously (20, 33, 44–48), multivalent display of RBDs on self-assembling protein nanoparticles improves immunogenicity, particularly post-prime, as indicated by the enhanced immunogenicity of the adjuvanted protein RBD nanoparticles compared with non-assembling RBD trimers at equivalent doses of RBD. Third, mRNA-launched RBD nanoparticles likely assemble properly in vivo, as indicated by the enhanced immunogenicity of the mRNA-launched RBD nanoparticles compared with non-assembling RBD trimers at equivalent doses of mRNA. Fourth, mRNA-launched RBD nanoparticles are as, if not more, immunogenic than adjuvanted two-component RBD nanoparticle protein vaccines, depending on the dose of mRNA. Finally, mRNA-launched RBD nanoparticles are several-fold more immunogenic than COVID-19 mRNA vaccines encoding membrane-anchored S-2P or a secreted RBD trimer. In summary, multivalent antigen display on computationally designed protein nanoparticles enhances immunogenicity, whether delivered as adjuvanted protein or mRNA vaccines.

### Rpk9-I3–01NS induced antigen-specific T cell responses

**Figure 3.**
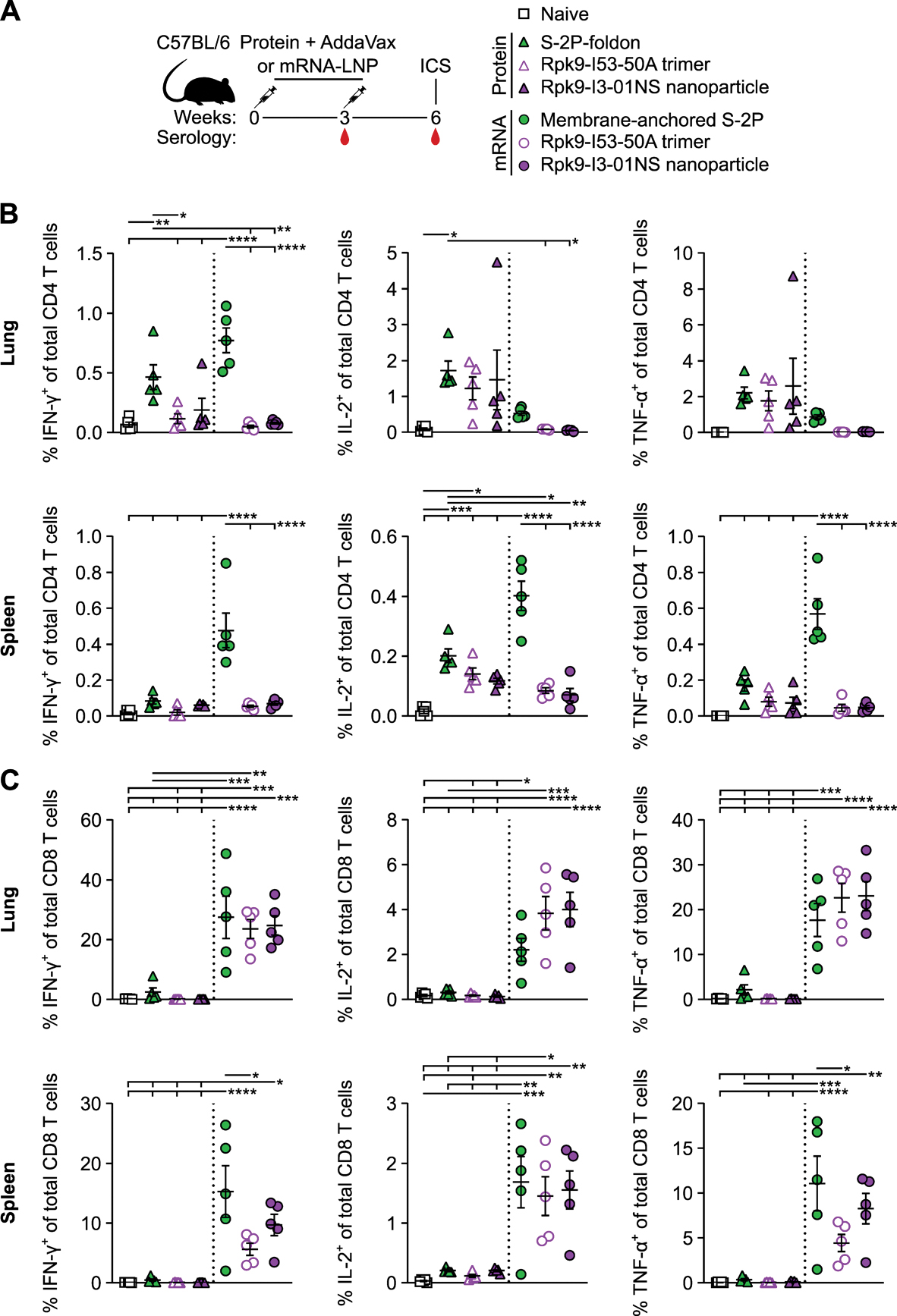
Membrane-anchored S-2P, Rpk9-I53–50A, and Rpk9-I3–01NS mRNA vaccines induce antigen-specific CD8 T cell responses in C57BL/6 mice. (A) Study design and groups (ICS, intracellular cytokine staining); n=5 mice/group received either AddaVax-adjuvanted protein (equimolar amounts of RBD: 0.9 μg of RBD per dose for Rpk9-based constructs, 5 μg of spike protein per dose for S-2P-foldon) or nucleoside-modified, LNP-encapsulated mRNA (1 μg per dose). (B and C) Percentage of CD4 T cells (B) and CD8 T cells (C) isolated from the lung or spleen that produced IFN-γ, IL-2, or TNF-α in response to ex vivo stimulation with a Wuhan-Hu-1 SARS-CoV-2 spike protein peptide pool of overlapping 15-mers. In (B and C), each symbol represents an individual animal. Error bars represent mean ± SEM. The dotted vertical line separates the protein and mRNA immunized groups. Statistical significance was determined using one-way ANOVA with Tukey’s multiple comparisons test; *p < 0.05; **p < 0.01; ***p < 0.001; ****p < 0.0001.

We next evaluated the antigen-specific T cell response induced by Rpk9-I3–01NS when delivered as an adjuvanted protein vaccine and an mRNA vaccine. As before, we also evaluated protein-delivered S-2P-foldon trimers and Rpk9-I53–50A trimers, as well as mRNA-delivered membrane-anchored S-2P trimers and secreted Rpk9-I53–50A trimers. Groups of five C57BL/6 mice were immunized intramuscularly at weeks 0 and 3 with either AddaVax-adjuvanted protein (equimolar amounts of RBD: 0.9 μg of RBD per dose for Rpk9-based constructs, 5 μg of spike protein per dose for S-2P-foldon) or nucleoside-modified, LNP-encapsulated mRNA (1 μg dose) (Fig. 3A). Three weeks post-boost, lung and spleen lymphocytes were isolated and stimulated in vitro with a peptide pool containing overlapping peptides from the Wuhan-Hu-1 SARS-CoV-2 spike protein. Following stimulation, intracellular staining was performed to detect the frequency at which individual cytokines were produced (fig. S6 to S8). For a baseline, we also isolated and stimulated lung and spleen lymphocytes from naive mice. As expected, we observed minimal to undetectable antigen-specific CD4 or CD8 T cells in either the lungs or spleens of these mice (Fig. 3, B and C).

In the lungs, mRNA-delivered membrane-anchored S-2P induced the highest frequency of interferon (IFN)-γ-expressing CD4 T cells (mean of 0.8% IFN-γ^+^ of total CD4 T cells) (p < 0.0001 for all comparisons, except for S-2P-foldon, where p = 0.051), whereas the frequencies of interleukin (IL)-2- and tumor necrosis factor (TNF)-α-expressing CD4 T cells were largely comparable across groups (Fig. 3B). In the spleen, mRNA-delivered membrane-anchored S-2P induced the highest frequencies of IFN-γ−, IL-2-, and TNF-α-expressing CD4 T cells among all groups (means of 0.5% IFN-γ^+^, 0.4% IL-2^+^, and 0.6% TNF-α^+^ of total CD4 T cells) (p < 0.0001 for all comparisons). With regard to antigen-specific CD8 T cell responses, all three mRNA vaccines induced comparable frequencies of IFN-γ, IL-2, and TNF-α-expressing CD8 T cells in the lungs (means ranging from 24–27% IFN-γ^+^, 2–4% IL-2^+^, and 18–23% TNF-α^+^ of total CD8 T cells), as well as comparable frequencies of IL-2-expressing CD8 T cells in the spleen (means ranging from 1.5–1.7% IL-2^+^ of total CD8 T cells) (Fig. 3C). However, mRNA-delivered membrane-anchored S-2P induced higher frequencies of IFN-γ− and TNF-α-expressing CD8 T cells in the spleen (means of 15% IFN-γ^+^ and 11% TNF-α^+^ of total CD8 T cells) compared with secreted Rpk9-I53–50A (p < 0.05 for both comparisons). All three adjuvanted protein vaccines induced minimal or undetectable antigen-specific CD8 T cell responses in either the lungs or spleen.

We note that our use of a peptide pool spanning the spike protein, rather than a pool matched to each full-length immunogen, means we have not captured the whole T cell response induced by the RBD nanoparticles and RBD trimers, as we have previously shown that computationally designed nanoparticle scaffolds themselves contain T cell epitopes (49). Further, spike protein-based immunogens comprise all of the possible T cell epitopes in the overlapping peptide library we used for stimulation, whereas the RBD-based immunogens comprise only a fraction of the epitopes ((50, 51)). Nonetheless, these results still corroborate previous studies in showing that mRNA vaccines induce substantially more robust CD8 T cell responses when compared with protein-delivered vaccines, even in the presence of adjuvants (29). We also note that the high frequencies of CD8 T cells elicited by the mRNA vaccines studied here are similar to those elicited by licensed COVID-19 vaccines in previous studies in mice (52) but that the frequencies observed in humans from the same vaccines were considerably lower (13).

In addition to evaluating the T cell response, we also performed serological analyses of the immunized groups three weeks post-prime and -boost to evaluate immunogenicity in a second mouse model (fig. S9 and S10). The results were generally consistent with those observed in BALB/c mice (Fig. 2, B and C, and fig. S2 and S3). However, in C57BL/6 mice, there appeared to be a smaller enhancement of immunogenicity provided by the mRNA-launched RBD nanoparticles.

### mRNA-launched Rpk9-I3–01NS conferred protection against challenge with mouse-adapted Wuhan-Hu-1 SARS-CoV-2

**Figure 4.**
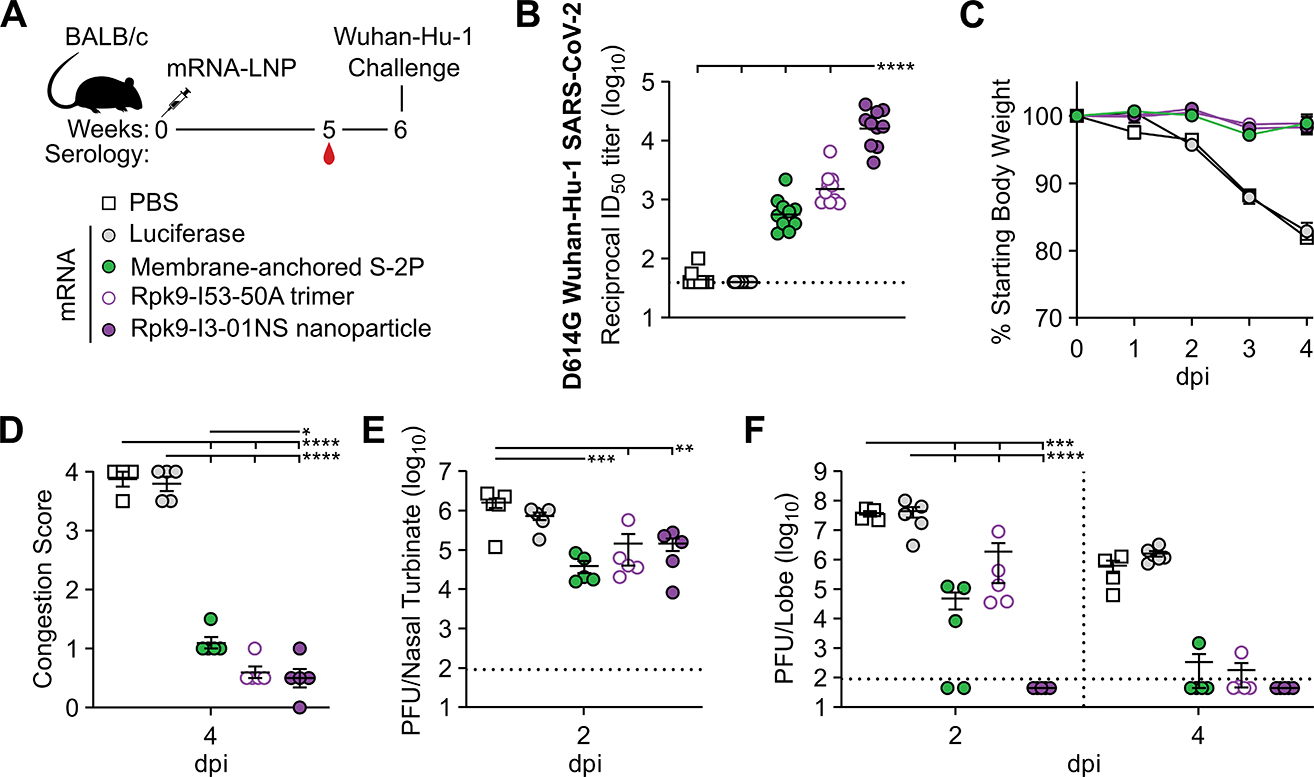
mRNA-launched Rpk9-I3–01NS elicits neutralizing activity and confers protective immunity against mouse-adapted Wuhan-Hu-1 SARS-CoV-2. (A) Study design and groups; n=4–6 mice/group/time point (2 and 4 days post infection (dpi)) received either nucleoside-modified, LNP-encapsulated mRNA (1 μg dose) or an equivalent volume of PBS. (B) Serum neutralizing antibody titers against D614G Wuhan-Hu-1 SARS-CoV-2 authentic virus. Each symbol represents an individual animal and the GMT from each group is indicated by a horizontal line. The dotted horizontal line represents the lowest limit of detection for the assay. (C) Weight loss up to 4 days dpi. Each symbol is the mean of the group for the time point ± SEM. Statistical significance has been omitted for simplicity but can be found in data file S3. (D) Congestion score at 4 dpi (scored as: 0 = no discoloration to 4 = severe discoloration). (E) Infectious viral load at 2 dpi in the nasal cavity after challenge of vaccinated mice as determined by plaque assay. (F) Infectious viral load at 2 and 4 dpi in the lung after challenge of vaccinated mice as determined by plaque assay. The dotted vertical line separates time points. The dotted horizontal line indicates the limit of detection; for samples with values below this, data are plotted at half the limit of detection. For (D to F), each symbol represents an individual animal. Error bars represent mean ± SEM. Statistical significance was determined using one-way (B, D, E) or two-way (F) ANOVA followed by Tukey’s multiple comparisons test. Weight loss data in (C) was analyzed using mixed effect analysis followed by Tukey’s multiple comparisons test. For all statistical analyses; *p < 0.05, **p < 0.01, ***p < 0.001, and ****p < 0.0001.

We next evaluated the protective efficacy of mRNA-launched Rpk9-I3–01NS nanoparticles compared with mRNA-delivered membrane-anchored S-2P trimers and secreted Rpk9-I53–50A trimers. We also evaluated an mRNA vaccine encoding luciferase as a control. BALB/c mice were immunized intramuscularly with either nucleoside-modified, LNP-encapsulated mRNA (1 μg dose) or an equivalent volume of phosphate-buffered saline (PBS) (Fig. 4A). Five weeks after the single immunization, vaccine-elicited antibody responses were assessed by serum ELISAs and neutralization assays with vaccine-matched (D614G Wuhan-Hu-1 SARS-CoV-2) authentic virus. Regardless of the ELISA antigen used (Wuhan-Hu-1 SARS-CoV-2 S-2P, HexaPro, or Rpk9-HexaPro), mRNA-launched Rpk9-I3–01NS elicited ≥5-fold higher antigen-specific binding antibody titers than mRNA-delivered membrane-anchored S-2P and secreted Rpk9-I53–50A (p < 0.0001 for all comparisons) (fig. S11). mRNA-launched Rpk9-I3–01NS also elicited 28-fold and 11-fold higher vaccine-matched neutralizing antibody titers than mRNA-delivered membrane-anchored S-2P and secreted Rpk9-I53–50A, respectively (p < 0.0001 for both comparisons) (Fig. 4B and fig. S12). As expected, immunization with PBS and luciferase mRNA elicited little to no vaccine-matched neutralization. One week later (i.e., six weeks after the single immunization), the mice were challenged intranasally with 1×10^5^ plaque-forming units (PFUs) of mouse-adapted Wuhan-Hu-1 SARS-CoV-2 MA10 (53) and followed for four days to assess protection from disease. All mice immunized with mRNA vaccines encoding SARS-CoV-2 immunogens were protected against weight loss and death for four days post infection (Fig. 4C). Contrarily, PBS- and luciferase mRNA-immunized mice experienced weight loss up to 20% of their starting body weight and one mouse from each group had succumbed to infection by the fourth day. Consistent with these data, we observed severe lung discoloration in PBS and luciferase mRNA-immunized mice four days post infection, an indication of severe disease, particularly inflammation, edema, and diffuse alveolar damage (Fig. 4D). By contrast, mice immunized with mRNA encoding SARS-CoV-2 immunogens showed minimal to no lung discoloration 4 days after infection (p < 0.0001 for all comparisons with PBS and luciferase). Furthermore, we observed significantly lower congestion scores for the mRNA-launched Rpk9-I3–01NS group compared with the membrane-anchored S-2P group (p < 0.05).

Analysis of viral titers in nasal turbinates and lung tissue further elucidated the performance of the mRNA vaccines in the upper and lower respiratory tract, respectively. Two days post infection, we observed viral loads in nasal turbinates across all groups (means ranging from 3.9×10^4^–1.6×10^6^ PFU/tissue)(Fig. 4E). These results are consistent with previous reports suggesting that prevention of viral replication in the upper respiratory tract can be difficult to achieve by intramuscular vaccination (54). Nonetheless, two days post infection, when viral replication of mouse-adapted Wuhan-Hu-1 SARS-CoV-2 MA10 has been shown to reach its peak in the lung (53), mRNA-launched Rpk9-I3–01NS provided complete protection from detectable virus in the lung (Fig. 4F). Contrarily, all other groups had detectable viral loads (means ranging from 4.9×10^4^–4.4×10^7^ PFU/tissue). These differences in lung viral titers at two days post infection are consistent with our authentic virus neutralization data, and align with previous reports showing that antigen-specific binding and vaccine-matched neutralizing antibody titers are correlates of protection against SARS-CoV-2 (55). Four days post infection, we observed a decrease in lung viral titers across all groups. All five mice immunized with mRNA-launched Rpk9-I3–01NS maintained undetectable viral loads in the lung and most mice immunized with mRNA-delivered membrane-anchored S-2P and secreted Rpk9-I53–50A had undetectable viral loads at this time point. By contrast, the PBS- and luciferase mRNA-immunized groups had detectable viral loads (means of 5.4×10^5^ and 1.6×10^6^ PFU/tissue, respectively). Altogether, these results indicate that mRNA-launched RBD nanoparticles elicit protective antibodies and provide protection against viral replication in the lung during vaccine-matched infection.

### Protection against challenge with mouse-adapted Omicron BA.5 SARS-CoV-2

**Figure 5.**
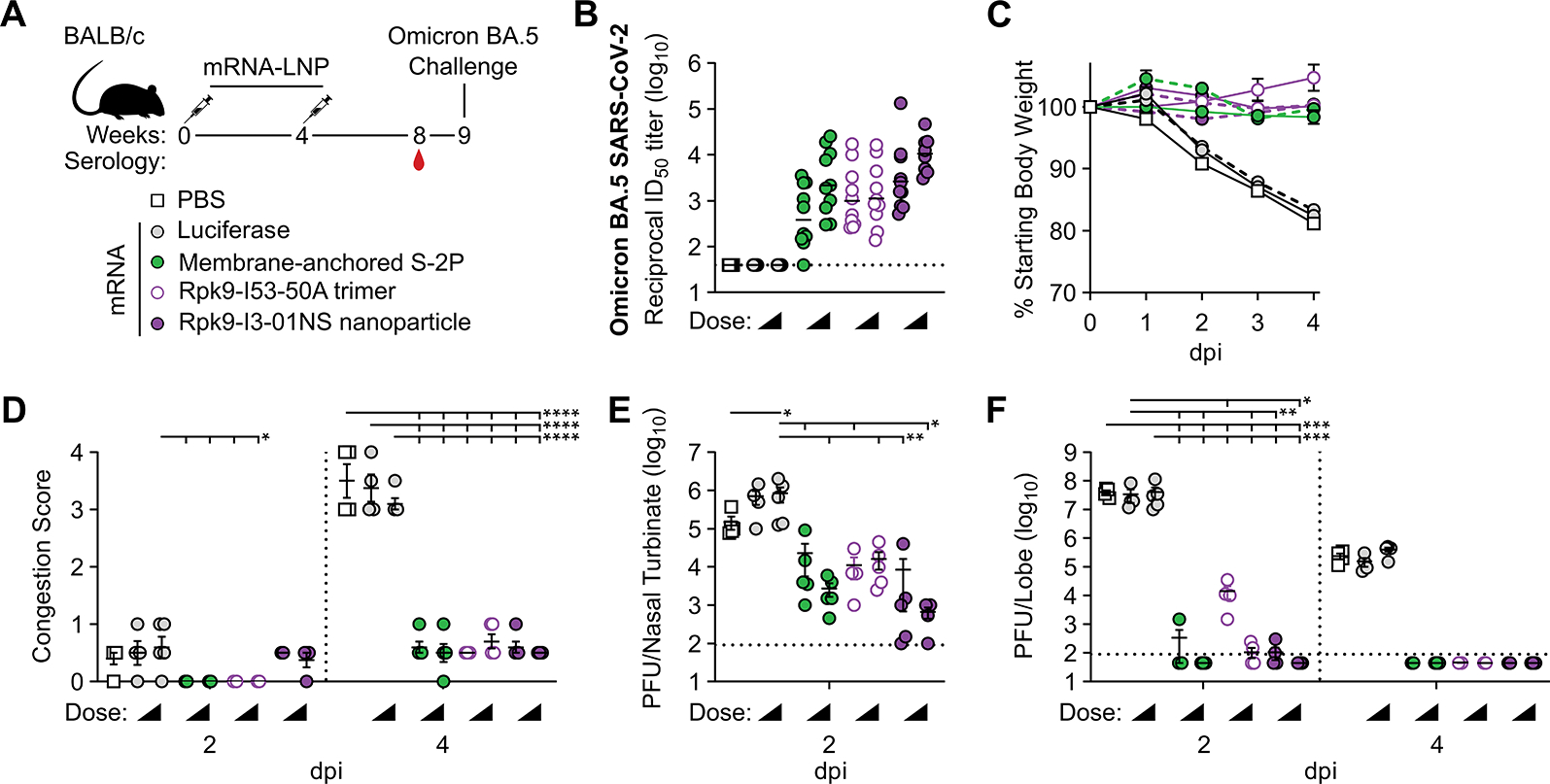
mRNA-launched Rpk9-I3–01NS elicits neutralizing activity and confers protective immunity against mouse-adapted Omicron BA.5 SARS-CoV-2. **(A)** Study design and groups; n=4–5 mice/group/time point (2 and 4 days post infection (dpi)) received either nucleoside-modified, LNP-encapsulated mRNA (1 or 5 μg dose) or an equivalent volume of PBS. **(B)** Serum neutralizing antibody titers against Omicron BA.5 SARS-CoV-2 authentic virus. Each symbol represents an individual animal and the GMT from each group is indicated by a horizontal line. The dotted horizontal line represents the lowest limit of detection for the assay. **(C)** Weight loss up to 4 dpi. Each symbol is the mean of the group for the time point ± SEM. The solid lines correspond to groups immunized with 1 μg of mRNA; the dashed lines correspond to groups immunized with 5 μg of mRNA. Statistical significance has been omitted for simplicity but can be found in data file S3. **(D)** Congestion score at 2 and 4 dpi (scored as in Fig. 4D). The dotted vertical line separates time points. **(E)** Infectious viral load at 2 dpi in the nasal cavity after challenge of vaccinated mice as determined by plaque assay. The dotted horizontal line indicates the limit of detection. **(F)** Infectious viral load at 2 and 4 dpi in the lung after challenge of vaccinated mice as determined by plaque assay. The dotted vertical line separates time points. The dotted horizontal line indicates the limit of detection; for samples with values below this, data are plotted at half the limit of detection. For (D to F), each symbol represents an individual animal. Error bars represent mean ± SEM. Statistical significance was determined using one-way (B, E) or two-way (D, F) ANOVA followed by Tukey’s multiple comparisons test. Weight loss data in (C) was analyzed using mixed effect analysis followed by Tukey’s multiple comparisons test. For all statistical analyses; *p < 0.05, **p < 0.01, ***p < 0.001, and ****p < 0.0001.

Next, we performed a vaccine-mismatched challenge study to evaluate the breadth of protection from mRNA-launched Rpk9-I3–01NS nanoparticles compared with mRNA-delivered membrane-anchored S-2P trimers and secreted Rpk9-I53–50A trimers. We again included an mRNA vaccine encoding luciferase as a control. BALB/c mice were immunized intramuscularly at weeks 0 and 4 with either nucleoside-modified, LNP-encapsulated mRNA (1 or 5 μg dose) or an equivalent volume of PBS (Fig. 5A). At week 8, (i.e., four weeks post-boost immunization), we observed vaccine-elicited antibody responses for all groups immunized with mRNA encoding SARS-CoV-2 immunogens, as assessed by serum neutralization assays with vaccine-mismatched (Omicron BA.5 SARS-CoV-2) authentic virus (Fig. 5B and fig. S13). At week 9 (i.e., five weeks post-boost), the mice were challenged intranasally with 1×10^5^ PFUs of mouse-adapted Omicron BA.5 SARS-CoV-2 MA10 (56) and followed for four days to assess protection from disease, as assessed by weight loss, congestion score, and viral titers.

All mice immunized with mRNA vaccines encoding SARS-CoV-2 immunogens were protected against weight loss and death for four days post infection, irrespective of dose of mRNA (Fig. 5C). Contrarily, mice immunized with PBS or mRNA encoding luciferase experienced weight loss up to 20% of their starting body weight. Further, one PBS-immunized mouse and one luciferase mRNA-immunized mouse (from the 1 μg dose group) had succumbed to infection by the fourth day. Consistent with these data, mice immunized with mRNA encoding SARS-CoV-2 immunogens showed minimal lung discoloration four days post infection compared with mice immunized with PBS and luciferase mRNA (p < 0.0001 for all comparisons) (Fig. 5D). Two days post infection, we observed viral loads in the nasal turbinates for all groups (means ranging from 6.6×10^2^–8.5×10^5^ PFU/tissue) (Fig. 5E). However, two mice immunized with mRNA-launched Rpk9-I3–01NS (one per dose of mRNA) were completely protected from detectable virus in the nasal turbinates. At the same time point, groups immunized with mRNA encoding SARS-CoV-2 immunogens had either undetectable or low viral loads in the lungs (means ranging from 1.1×10^2^–1.5×10^4^ PFU/tissue), whereas PBS- and luciferase mRNA-immunized groups had high viral loads (means ranging from 3.4×10^7^–4.1×10^7^ PFU/tissue) (p < 0.001 when comparing the PBS and 5 μg luciferase groups with all other mRNA groups; p < 0.01 or 0.05 when comparing the 1 μg luciferase group with all other mRNA groups) (Fig. 5F). Two days later (i.e., four days post infection), we observed a decrease in lung viral titers across all groups and that all groups immunized with mRNA encoding SARS-CoV-2 immunogens had undetectable viral loads, irrespective of mRNA dose. Altogether, these results indicate that mRNA-launched RBD nanoparticles provide protection against vaccine-mismatched challenge.

### mRNA-launched nanoparticles offer a versatile vaccine platform

Intrigued by the successful secretion of Rpk9-I3–01NS, but not RBD-I3–01NS, we next sought to better understand the relationships between the displayed antigen, the nanoparticle scaffold, and secretion, and to evaluate the generalizability of our technology platform beyond Wuhan-Hu-1 SARS-CoV-2. To assess the relationship between the nanoparticle scaffold and secretion, we selected ferritin as a comparator nanoparticle scaffold and evaluated RBD-Ferritin and Rpk9-Ferritin fusion constructs (table S1). Following plasmid DNA transfection of human (Expi293F) cells, SDS-PAGE of cell culture pellets and supernatants revealed that both RBD-Ferritin and Rpk9-Ferritin constructs expressed, but only Rpk9-Ferritin secreted (fig. S14A). These observations were consistent with our initial evaluation of RBD-I3–01NS and Rpk9-I3–01NS (fig. S1A to D). Further purification of Rpk9-Ferritin by SEC revealed a predominant peak corresponding to the target octahedral assembly (fig. S14B), and DLS and nsEM confirmed a homogenous and monodisperse population of nanoparticles (fig. S14, C and D). Lastly, BLI confirmed the antigenic integrity of Rpk9-Ferritin by demonstrating binding to hACE2-Fc, CR3022, and S309 (fig. S14E). These data indicate that Rpk9-Ferritin, but not RBD-Ferritin, nanoparticles secrete efficiently and are biochemically, antigenically, and structurally intact.

To further assess the relationship between the displayed antigen and secretion, we selected the antigenically distant Omicron BA.5 as a representative SARS-CoV-2 variant for multivalent display on both I3–01NS and ferritin nanoparticles. Given the requirement for the Rpk9 mutations for successful secretion of Wuhan-Hu-1-displaying nanoparticles, as well as previously published data demonstrating the portability of Rpk9 mutations to other SARS-CoV-2 variants (57), we only evaluated BA.5-Rpk9-I3–01NS and BA.5-Rpk9-Ferritin fusion constructs (table S1). Following plasmid DNA transfection of human (Expi293F) cells, SDS-PAGE of cell culture pellets and supernatants revealed that both BA.5-Rpk9-Ferritin and BA.5-Rpk9-I3–01NS constructs expressed but failed to secrete (fig. S14A). These data, in addition to the Wuhan-Hu-1 data above on both I3–01NS and ferritin, indicate that the displayed antigen is a primary determinant of secretion.

**Figure 6.**
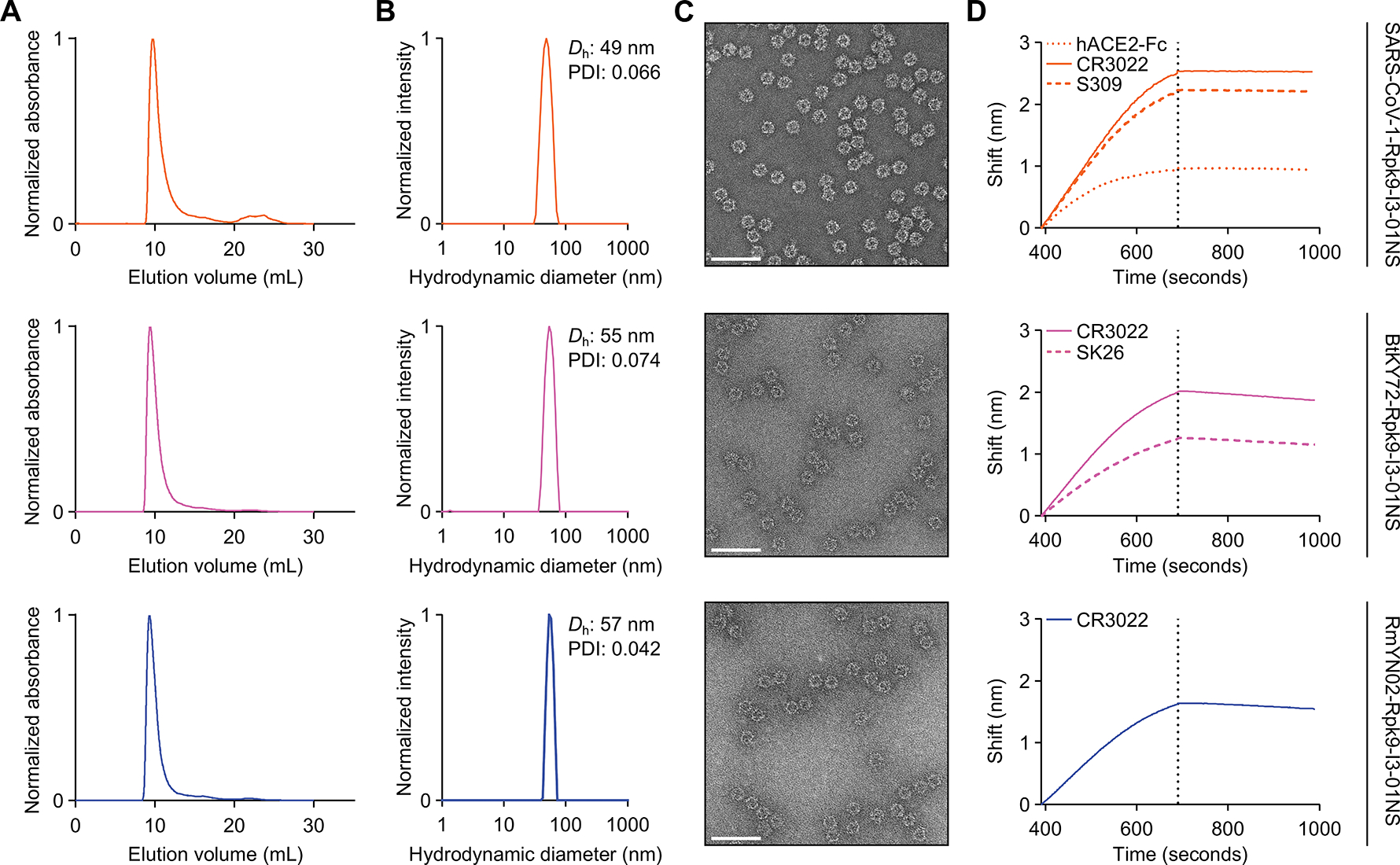
Characterization of secretable sarbecovirus RBD nanoparticles. **(A)** Size exclusion chromatogram of SARS-CoV-1-Rpk9-I3–01NS (orange), BtKY72-Rpk9-I3–01NS (pink), and RmYN02-Rpk9-I3–01NS (blue) purification. **(B)** DLS of SEC-purified SARS-CoV-1-Rpk9-I3–01NS, BtKY72-Rpk9-I3–01NS, and RmYN02-Rpk9-I3–01NS. *D*_h_, hydrodynamic diameter; PDI, polydispersity index. **(C)** Representative electron micrograph (scale bars = 100 nm) of negatively stained SEC-purified SARS-CoV-1-Rpk9-I3–01NS, BtKY72-Rpk9-I3–01NS, and RmYN02-Rpk9-I3–01NS. **(D)** Binding of immobilized hACE2-Fc, CR3022, S309, and SK26 to SEC-purified SARS-CoV-1-Rpk9-I3–01NS, BtKY72-Rpk9-I3–01NS, and RmYN02-Rpk9-I3–01NS as assessed by BLI. The dotted vertical line separates the association and dissociation steps.

Despite the inability to secrete BA.5-Rpk9-I3–01NS, we were nonetheless able to produce and characterize I3–01NS nanoparticles displaying antigens from four additional viruses (Fig. 6, fig. S15, and table S1). Motivated by recurrent zoonoses of ACE2-utilizing sarbecoviruses, the spillover potential of non-ACE2-utilizing sarbecoviruses, and our success with a clade 1b sarbecovirus (SARS-CoV-2), we selected one virus from each of the remaining sarbecovirus clades: SARS-CoV-1 from clade 1a, BtKY72 from clade 3, and RmYN02 from clade 2. Since the linoleic acid binding pocket where all three Rpk9 mutations reside is widely conserved across human coronaviruses (34), we focused solely on the evaluation of Rpk9-I3–01NS nanoparticles. Beyond sarbecoviruses, we also evaluated I3–01NS displaying eOD-GT8, a germline-targeting, engineered outer domain of HIV-1 gp120 (58). Human (Expi293F) cells were transfected with plasmid DNA encoding the SARS-CoV-1-Rpk9-I3–01NS, BtKY72-Rpk9-I3–01NS, RmYN02-Rpk9-I3–01NS, and eOD-GT8-I3–01NS fusion constructs. SDS-PAGE of cell culture supernatants revealed successful secretion of all four constructs (fig. S15A) and purification by SEC revealed predominant peaks corresponding to the target icosahedral assemblies (Fig. 6A, and fig. S15B). Additional biophysical analyses by DLS and nsEM confirmed homogenous and monodisperse populations of nanoparticles (Fig. 6, B and C, fig. S15, C and D). Lastly, we confirmed the antigenic integrity of each construct by measuring binding to a panel of mAbs and host receptor proteins using BLI (Fig. 6D, fig. S15E). Specifically, SARS-CoV-1-Rpk9-I3–01NS, BtKY72-Rpk9-I3–01NS, and RmYN02-Rpk9-I3–01NS all bound CR3022, a broadly cross-reactive sarbecovirus RBD-directed mAb (59), whereas eOD-GT8-I3–01NS bound three variants of VRC01, a CD4 binding site-directed, broadly neutralizing antibody (60). Furthermore, SARS-CoV-1-Rpk9-I3–01NS also bound hACE2-Fc and S309, whereas BtKY72-Rpk9-I3–01NS bound SK26, a recently described, RBD-directed BtKY72-specific mAb (61). Altogether, these data indicate that several antigen-displaying nanoparticles secrete efficiently and are biochemically, antigenically, and structurally intact, demonstrating the versatility of I3–01NS as a scaffold for mRNA-launched protein nanoparticle immunogens.

## Discussion

To develop our technology platform, we leveraged computational protein design to generate an RBD nanoparticle immunogen compatible with delivery as an mRNA vaccine. Specifically, we used a one-component (i.e., homomeric) protein nanoparticle designed for optimal secretion (30) to display a SARS-CoV-2 RBD antigen designed for improved stability and expression (33). When produced in cell culture, the RBD nanoparticle was found to efficiently secrete and self-assemble while maintaining its structure and antigenicity. When delivered as an mRNA vaccine, the RBD nanoparticle elicited neutralizing antibody responses with high potency, breadth, and protective capacity. Additionally, the RBD nanoparticle induced robust antigen-specific CD8 T cell responses when delivered as an mRNA vaccine, but not as an adjuvanted protein vaccine. These results highlight how computationally designed mRNA-launched protein nanoparticle immunogens retain the advantageous features of both adjuvanted protein nanoparticle vaccines (i.e., enhanced B cell activation and expansion (22), superior trafficking to lymph nodes and B cell follicles (23, 24)) and mRNA-LNP vaccines (i.e., intracellular processing and major histocompatibility complex class I presentation of antigen (62), inherent adjuvanticity of LNPs (15, 16, 52)).

Despite the immunological benefits and potential manufacturing advantages (i.e., rapid and sequence-invariant manufacturing of nucleic acids), the genetic delivery of protein nanoparticle immunogens is a relatively new approach to vaccine design. Moreover, the field to date has relied on naturally occurring self-assembling proteins as scaffolds (i.e., ferritin, lumazine synthase, VLPs) (25–27, 63–66). The one genetically delivered protein nanoparticle immunogen that has advanced to clinical trials, eOD-GT8 60mer (67), is based on lumazine synthase. Our study introduces I3–01NS to the field as a genetically deliverable, computationally designed protein nanoparticle scaffold. Going forward, we envision two trajectories for the genetic delivery of computationally designed protein nanoparticle immunogens, each with its respective advantages.

First, several non-secretion-optimized variants of I3–01NS (i.e., mi3, I3–01v9, and the original computationally designed I3–01) have previously been used as broadly applicable scaffolds to generate a variety of adjuvanted protein nanoparticle vaccine candidates (47, 68–72). I3–01NS may prove a similarly versatile scaffold in the context of genetically deliverable protein nanoparticle immunogens. Our data showing that I3–01NS displaying any of four sarbecovirus RBDs or eOD-GT8 secretes and forms structurally and antigenically intact nanoparticle immunogens supports this possibility. However, the failure of the Wuhan-Hu-1 RBD and BA.5-Rpk9 to secrete when displayed on either I3–01NS or ferritin indicates that further work will be required to fully understand and optimize the relationships between displayed antigens, nanoparticle scaffolds, secretion, and immunogenicity. Further to this point, despite the immense worldwide effort concentrated on SARS-CoV-2 since early 2020, we are aware of only two previous reports of the secretion of well-defined protein nanoparticles displaying SARS-CoV-2 RBDs, neither of which displayed the wild-type Wuhan-Hu-1 RBD; secreting the Wuhan-Hu-1 RBD on ferritin required engineering several N-linked glycans into the antigen, whereas the Delta RBD on ferritin secreted “with a very low yield” (26, 66). Our finding that the Rpk9 mutations were sufficient to enable robust secretion of the Wuhan-Hu-1 RBD on I3–01NS is a key takeaway from our work that should inform future vaccine design. With the right accompanying antigen design, I3–01NS-based mRNA-launched protein nanoparticle immunogens could become a modular platform for pandemic threats and rapidly mutating viruses.

Second, several recent studies have shown that the detailed geometry of antigen presentation can substantially impact B cell activation and vaccine-elicited antibody responses (22, 73, 74). Furthermore, there are several important classes of antigens, such as tetrameric influenza neuraminidases and paramyxovirus receptor-binding proteins, that lack nanoparticle scaffolds suitable for genetic fusion due to symmetry mismatches (75, 76). As such, relying on the relatively unalterable structural features of naturally occurring self-assembling proteins or I3–01NS alone is a considerable limitation. Computational methods that allow the design of self-assembling proteins with atomic-level accuracy may provide a route to overcoming this limitation by designing genetically deliverable protein nanoparticle immunogens with custom structural and functional features (77). The recent development of powerful machine learning-based methods for protein structure prediction and design (78–80) will facilitate these efforts and may enable the development of nanoparticle vaccine scaffolds that are tailored to specific antigens and satisfy the requirements of genetic delivery.

There are several limitations to our study. First, despite evaluating nanoparticle assembly and measuring the yield of secreted RBD nanoparticles in vitro, we are currently unable to do so in vivo. Additionally, we have not yet directly assessed where nanoparticle assembly is occurring within the cell, either in vitro or in vivo. Second, although we observed consistent vaccine-elicited immune responses across multiple mouse models, the ability of these models to predict the performance of mRNA-launched RBD nanoparticles in higher mammals, including humans, is currently unknown. Third, we do not currently have data on the biodistribution, pharmacokinetics, immunological mechanisms, and durability of the vaccine-elicited immune response of these mRNA-launched protein nanoparticle immunogens beyond the functional endpoints measured after immunization reported here. Such information will be helpful for guiding iterative improvement of the platform and advancing it to clinical development.

In conclusion, our work demonstrates the utility of, and lays the foundation for, computationally designed mRNA-launched protein nanoparticle immunogens as an integrated vaccine platform. We anticipate that this technology will be useful in designing vaccines against various viral, bacterial, and parasitic pathogens. Furthermore, this platform may be particularly valuable for pandemic prevention, preparedness, and response (81).

## Materials and Methods

### Study design

The overall objective was to evaluate a computationally designed protein nanoparticle immunogen when delivered as an mRNA vaccine. Cell culture was used to produce proteins for characterization and immunization. Four mouse studies were conducted to assess vaccine-elicited immune responses. The first study consisted of two separate arms, where four groups of BALB/c mice (n=10/group) were immunized twice with adjuvanted proteins and ten groups of BALB/c mice (n=10/group) were immunized twice with varying doses of mRNA-LNPs or empty LNP (n=5). Blood draws were conducted twice for downstream serological analyses. The second study consisted of seven groups of C57BL/6 mice (n=5/group), six of which were immunized twice with either adjuvanted proteins or mRNA-LNPs and one which was kept unimmunized as a negative control. Spleens and lungs were harvested for downstream T cell analyses. Blood draws were conducted twice for downstream serological analyses. The third study consisted of five groups of BALB/c mice (n=4–6 mice/group/time point), four of which received a single dose of mRNA-LNPs and one which received an equivalent volume of PBS. Blood draws were conducted for downstream serological analyses prior to lethal challenge with vaccine-matched mouse-adapted virus. Body weights were observed for up to four days after infection. Mice were euthanized either two or four days after infection, followed by the collection of lungs and nasal turbinates for downstream plaque analyses. The fourth study consisted of five groups of BALB/c mice (n=4–5 mice/group/time point), all of which received two doses of mRNA-LNPs. Blood draws were conducted for downstream serological analyses prior to lethal challenge with vaccine-mismatched mouse-adapted virus. Body weights were observed for up to four days after infection. Mice were euthanized either two or four days after infection, followed by the collection of lungs and nasal turbinates for downstream plaque analyses. Each mouse study included at least one negative control group (i.e., naive, luciferase mRNA, empty LNP, PBS). The immunization schedules and doses for each mouse study are provided in the main text and were determined on the basis of our previous experience, as well as prior literature. The number of animals in each group was determined to identify large differences between groups on the basis of our previous experience. We did not do a power calculation to determine the sample size. The animals were randomly distributed between groups. The investigators were not blinded during experiments and outcome assessment. The number of technical and experimental replicates are provided either in figure legends or separate methods sections.

### Statistical analysis

Source data for main text figures and supplemental figures are presented in data files S1 and S2, respectively. Statistical significance has been presented in figures unless otherwise stated in the figure legend. All statistical analyses are available in data file S3. Serological data, intracellular cytokine staining data, and nasal turbinate viral titer data were analyzed using one-way ANOVA followed by Tukey’s multiple comparisons test. Congestion scores were analyzed using one-way (Fig. 4D) or two-way (Fig. 5D) ANOVA followed by Tukey’s multiple comparisons test. Weight loss data was analyzed using mixed effect analysis followed by Tukey’s multiple comparisons test. Lung viral titer data was analyzed using two-way ANOVA followed by Tukey’s multiple comparisons test. For all statistical analyses, *p < 0.05, **p < 0.01, ***p < 0.001, and ****p < 0.0001.

## Supplementary Material

Supplemental Material

Data file S1

Data file S2

MDAR Reproducibility Checklist

Data file S3

List of Supplementary Materials

This PDF file includes:

Materials and Methods

Figs. S1 to S15

Tables S1 to S3

Other Supplementary Material for this manuscript includes the following:

Data files S1 to S3

MDAR Reproducibility Checklist

## Data Availability

All data associated with this study are in the paper or supplementary materials. All images and data were generated and analyzed by the authors. Source data are provided in data files S1 and S2. All reagents will be made available on request after completion of a Material Transfer Agreement.
