# Supplementary material for: Computationally designed mRNA-launched protein nanoparticle immunogens elicit protective antibody and T cell responses in mice
Source: Sci Transl Med. Author manuscript; Available in PMC 2026 Mar 30. (PMC13035345; doi:10.1126/scitranslmed.adu2085)
Supplement: Supplemental Material [file NIHMS2118270-supplement-Supplemental_Material.pdf]

Materials and Methods

Plasmid construction

Wild-type RBD, stabilized Rpk9, and eOD-GT8 were genetically fused to the N termini of I3-01NS and *Helicobacter pylori* ferritin using a 16-residue glycine/serine linker (GGSGGSGSGGSGGSGS). All sequences contained an N-terminal mu-phosphatase signal peptide (MGILPSPGMPALLSLVSLLSVLLMGCVAETGT), except for eOD-GT8-I3-01NS, which contained an N-terminal bovine prolactin signal peptide (MDSKGSSQKGSRLLLLLVVSNLLLPQGVLA). Sequences were codon-optimized for human cell expression and cloned into pCMV/R using the XbaI and AvrII restriction sites by Genscript. The plasmids for hACE2-Fc, CR3022, Rpk9-I53-50A, S-2P-foldon, HexaPro, Rpk9-HexaPro, I3-01NS, I53-50A, I53-50B, CV30, and SK26 were synthesized as previously described *(20, 30, 33, 37, 61, 84)*. Plasmids were transformed into the NEB 5α strain of *E. coli* (New England Biolabs) for subsequent DNA extraction from bacterial culture (QIAGEN Plasmid Plus Maxi Kit protocol) to obtain plasmid for transient transfection into Expi293F cells. The amino acid sequences of proteins described in this study can be found in **table S1**. The amino acid sequences of proteins used in immunizations can be found in **table S2**.

Protein production and purification

For purification of plasmid DNA for Expi293F transfection, bacteria were cultured and plasmids were harvested according to the QIAGEN Plasmid Plus Maxi Kit protocol (QIAGEN). Expi293F cells are derived from the HEK293F cell line, a female human embryonic kidney cell line transformed and adapted to grow in suspension. Expi293F cells were grown in suspension and passaged according to manufacturer protocols (Thermo Fisher). Cells at 3.0×10^{6} cells/mL were transfected with 1 µg of purified plasmid DNA mixed with 3 µg of PEI-MAX per mL cell culture. Supernatant was harvested 72 h post-transfection by centrifugation for 5 minutes at 4,100 *g* followed by the addition of PDADMAC solution (Sigma Aldrich), a second centrifugation for 5 minutes at 4,100 *g*, and sterile filtration. Cell pellets were washed twice then resuspended in the same volume of the removed medium with phosphate-buffered saline (PBS). Resuspended cells were adjusted to 0.05% Triton-X 100 and 50 units/mL Benzonase endonuclease (Millipore Sigma) followed by a 10 min incubation at 37°C.

To purify Rpk9-I3-01NS for animal studies, the supernatant was adjusted to 50 mM Tris (pH 8.0) and 150 mM NaCl. Galanthus Nivalis Gel (GNA) immobilized lectin conjugated resin (EY Laboratories) was rinsed into magnesium- and calcium-free Dulbecco's phosphate buffered saline (DPBS) (Gibco) using a gravity column and then added to the supernatant, followed by overnight shaking at 4°C. The resin was collected using a gravity column and washed with Lectin Wash Buffer (50 mM Tris (pH 8.0), 150 mM NaCl, 100 mM Arginine (pH 8.0), 5% (v/v) glycerol, and 0.02% (w/v) NaN_3). Protein was eluted using Lectin Elution Buffer (50 mM Tris (pH 8.0) 150 mM NaCl, 100 mM Arginine (pH 8.0), 5% (v/v) glycerol, 0.02% (w/v) NaN_3, and 1 M Methyl-α-D-mannopyranoside). Eluates were concentrated and applied to a Superose 6 Increase 10/300 GL column pre-equilibrated with Sizing Buffer (50 mM Tris (pH 8.0), 150 mM NaCl, 100 mM Arginine (pH 8.0), and 5% (v/v) glycerol) for preparative SEC. Peaks corresponding to RBD nanoparticles were identified based on elution volume, pooled together, and sterile-filtered. Protein samples were stored at 4°C or -80°C until use. To purify Rpk9-I3-01NS for supernatant ELISA standard curves, the 5% (v/v) glycerol and 0.02% (w/v) NaN_3 were omitted from the Lectin Wash Buffer and Lectin Elution Buffer. Purification of Rpk9-Ferritin, SARS-CoV-1-Rpk9-I3-01NS, BtKY72-Rpk9-I3-01NS, RmYN02-Rpk9-I3-01NS, and eOD-GT8-I3-01NS was performed similarly.

Purification of hACE2-Fc, CR3022, Rpk9-I53-50A, S-2P-foldon, HexaPro, Rpk9-HexaPro, I3-01NS, I53-50A, I53-50B, CV30, and SK26 was performed as previously described *(20, 30, 33, 37, 61, 84)*. Purified S309 was

commercially purchased from Abcam (ab289796). HIV Env-specific antibodies were synthesized, expressed in CHO cells, and purified using protein A by Genscript. The three VRC01 variants were mature VRC01, an inferred germline (iGL) version with germline-reverted variable and joining genes but with a mature HCDR3, and an unmutated common ancestor (UCA) variant with known HCDR3 and LCDR3 recombination mutations as well as a reversion of a known deletion in the LCDR1 *(85, 86)*.

Ultraviolet-visible spectrophotometry (UV/vis)

Protein samples were applied to a 10 mm, 50 µL quartz cell (Starna Cells, Inc.) and absorbance was measured from 180 to 1000 nm using an Agilent Cary 8454 spectrophotometer. Net absorbance at 280 nm, obtained from measurement and single reference wavelength baseline subtraction, was used with calculated extinction coefficients and molecular weights to obtain protein concentration. Samples were diluted with respective blanking buffers to obtain an absorbance between 0.1 and 1.0. All data produced from the UV/vis instrument was processed in the 845x UV/visible System software.

Dynamic light scattering (DLS)

Hydrodynamic diameter (D_h) and polydispersity index (PDI) was measured on an UNcle Nano-DSF (UNchained Laboratories) at 25°C. Sample was applied to a 8.8 µL quartz capillary cassette (UNi, UNchained Laboratories) and measured with 10 acquisitions of 5 s each, using auto-attenuation of the laser. Protein concentration (ranging from 0.1–1.0 mg/mL) and increased viscosity due to the inclusion of 5% v/v glycerol in the buffer was accounted for by the UNcle Client software.

Negative stain electron microscopy (nsEM)

Protein samples (3–6 µL, 0.05–0.1 mg/mL) were applied to glow-discharged 300-mesh copper grids (Ted Pella) and stained with uranyl formate (0.75–2% (w/v)). Data were collected using a 120 kV Talos L120C transmission electron microscope (Thermo Scientific) with a BM-Ceta camera. CryoSPARC *(87)* was used for CTF correction, particle picking and extraction, and 2D classification.

Biolayer interferometry (BLI)

Binding of Fc-tagged receptors and IgGs to antigen-displaying nanoparticles was analyzed using an Octet Red 96 System (Pall FortéBio/Sartorius) at ambient temperature with shaking at 1000 rpm. Protein samples were diluted to 100 nM in Sartorius Octet Kinetics Buffer 10× (diluted to 1×). Kinetics Buffer, antibody, receptor, and immunogen were then applied to a black 96-well Greiner Bio-one microplate at 200 µL per well. Protein A biosensors were first hydrated for 10 min, then equilibrated in Kinetics Buffer for 30 s. The tips were then dipped into Fc-tagged receptors and IgGs diluted to 10 µg/mL in Kinetics Buffer for 300 s, then transferred back into Kinetics Buffer for 60 s to reach a baseline. The association step was performed by dipping the loaded biosensors into the immunogens for 300 s, and the subsequent dissociation step was performed by dipping the biosensors back into the Kinetics Buffer used to baseline for an additional 300 s. The data were baseline subtracted prior to plotting using the FortéBio analysis software.

Supernatant enzyme-linked immunosorbent assays (ELISA)

Supernatants were serially diluted 1:2 in Expi293 Expression Medium (Gibco). Purified protein (Rpk9-I3-01NS for RBD nanoparticles; Rpk9-I53-50A for RBD trimers) was diluted in Expi293 Expression Medium (Gibco) to create a standard curve at known concentrations. For each immunogen, 100 µL of the cognate supernatant and standard curve dilutions were plated onto 96-well Nunc Maxisorp (Thermo Fisher) plates. Plates were incubated at 25°C for 1 h then washed 3× using a plate washer (BioTek). Plates were blocked with 200 µL of 5% non-fat

milk in TBST (25 mM Tris (pH 8.0), 150 mM NaCl, 0.05% (v/v) Tween-20) for 1 h at 25°C. Plates were washed 3× in TBST, then 100 µL of 4 µg/mL CV30 *(88)*, a RBD-directed mAb, was added to each well and incubated at 25°C for 1 h. Plates were washed 3× in TBST, then goat anti-human IgG horseradish peroxidase-conjugated antibodies (Southern Biotech #2040-05) were diluted 1:5,000 and 100 µL was added to each well and incubated at 25°C for 30 min. Plates were washed 3× in TBST and 100 µL of TMB (SeraCare) was added to every well for 2 min at room temperature. The reaction was quenched with the addition of 100 µL of 1 N HCl. Plates were immediately read at 450 nm on an Epoch2 plate reader. The data was plotted and fit in Prism (GraphPad) using a four parameter logistic curve to determine the concentration of secreted protein.

Endotoxin measurements
Endotoxin concentrations in protein samples were measured using the EndoSafe PTS System (Charles River). Samples were diluted 1:50 or 1:100 in Endotoxin-free LAL reagent water, and applied into wells of an EndoSafe LAL reagent cartridge. Endotoxin values were reported as EU/mL with the dilution factor automatically back-calculated. Our threshold for samples suitable for immunization was <5 EU/dose. Protein samples with endotoxin concentrations above this threshold were adjusted to 0.75% w/v 3-[(3-cholamidopropyl)dimethylammonio]-1-propanesulfonate (CHAPS) and dialyzed three times against respective Sizing Buffers in a hydrated 20K molecular weight cutoff dialysis cassette (Thermo Fisher).

mRNA design and in vitro transcription
Membrane-anchored S-2P mRNA was designed to be identical to the publicly available, reverse engineered nucleic acid sequence of Pfizer/BioNTech's BNT162b2 vaccine *(41, 42)*. Rpk9-I53-50A and Rpk9-I3-01NS mRNAs were designed by retaining the UTRs and polyA tail from the Pfizer/BioNTech BNT162b2 vaccine but replacing the spike protein-encoding open-reading frame (ORF) for the codon-optimized ORF of the Rpk9-I53-50A and Rpk9-I3-01NS pCMV/R expression plasmids, respectively. In vitro transcription of membrane-anchored S-2P, Rpk9-I3-01NS, and Rpk9-I53-50A mRNA was performed by TriLink Biotechnologies using previously described standard protocols *(8, 89)*, N1-methylpseudouridine-5'-triphosphate (m1Ψ-5′-triphosphate) (TriLink #N-1081) instead of uridine-5'-triphosphate (UTP), and the CleanCap Reagent AG (TriLink #N-7113) for co-transcriptional capping. The firefly luciferase (Luc)-encoding mRNA was produced as described *(90)*. Briefly, the Luc sequence was codon-optimized, synthesized, and cloned into an mRNA production plasmid. The mRNA was transcribed to contain a 101 nucleotide-long poly(A) tail. N1-methylpseudouridine-5'-triphosphate (m1Ψ-5′-triphosphate) (TriLink #N-1081) instead of uridine-5'-triphosphate (UTP) was used to generate modified nucleoside-containing mRNA. Capping of the in vitro transcribed mRNAs was performed co-transcriptionally using the trinucleotide cap1 analog, CleanCap (TriLink #N-7413). mRNA was purified by cellulose (Sigma-Aldrich #11363-250G) purification. Luciferase mRNAs were analyzed by agarose gel electrophoresis and were stored frozen at −20 °C. Nucleic acid sequences for the open reading frames (ORFs) of mRNA constructs used for immunizations can be found in **table S3**.

Lipid nanoparticle (LNP) encapsulation of mRNAs
LNPs used in this study were similar in composition to those previously described *(91, 92)* and contain ionizable lipids proprietary to Acuitas Therapeutics (pKa ranging from 6.0–6.5). Nucleoside-modified mRNAs were encapsulated in LNPs using a self-assembly process in which an aqueous solution of mRNA (pH 4.0) was rapidly mixed with an aqueous solution of lipids (ionizable lipid/distearoylphosphatidylcholine (DSPC)/cholesterol/PEGylated lipid) dissolved in ethanol. The resultant mRNA-LNPs were characterized at Acuitas Therapeutics for their size, polydispersity, and encapsulation efficiency, and stored at -80°C until use. All mRNA-LNPs were sterile-filtered prior to vialing and freezing.

Formulation of proteins and mRNA-LNPs for immunizations

Prior to all immunizations, protein immunogen suspensions were diluted in Sizing Buffer and gently mixed 1:1 (v/v) with AddaVax adjuvant (Invivogen) to reach a final dose of 0.9 µg of RBD per mouse for Rpk9-based constructs or 5 µg of spike protein per mouse for S-2P-foldon, which comprise equimolar amounts of RBD antigen. Prior to all immunizations, mRNA-LNP suspensions were diluted with magnesium- and calcium-free DPBS (Gibco) to reach a final dose of 0.2, 1, or 5 µg mRNA per mouse.

Protein and mRNA-LNP immunogenicity study

Female BALB/c mice were purchased from Envigo (order code 047) at 7 weeks of age and were maintained in a specific pathogen-free facility within the Department of Comparative Medicine at the University of Washington, Seattle, accredited by the Association for Assessment and Accreditation of Laboratory Animal Care (AAALAC). Animal experiments were conducted in accordance with the University of Washington's Institutional Animal Care and Use Committee under protocol 4470-01. Mice of 8 weeks of age were injected intramuscularly into the quadriceps muscle of both hind legs with 50 µL per injection site under isoflurane anesthesia. Blood was collected by submental venous puncture and rested in serum separator tubes (BD # 365967) at room temperature for 30 min to allow for coagulation. Serum was separated from hematocrit by centrifugation at 2,000 *g* for 10 min. Complement factors and pathogens in isolated serum were heat-inactivated by incubation at 56°C for 60 min in a Bio-Rad T100 Thermal Cycler. Serum was stored at 4°C or -80°C until use.

Serum enzyme-linked immunosorbent assays (ELISA)

For antigen-specific ELISAs, 100 µL of 2 µg/mL Wuhan-Hu-1 SARS-CoV-2 S-2P, HexaPro, or Rpk9-HexaPro was plated onto 96-well Nunc Maxisorp (Thermo Fisher) plates in 50 mM Tris (pH 8.0), 150 mM NaCl, 0.25% (v/v) L-Histidine, 5% (v/v) glycerol. For scaffold-specific ELISAs, 100 µL of 2 µg/mL I3-01NS, I53-50A, or I53-50 was plated onto 96-well Nunc Maxisorp (Thermo Fisher) plates in TBS (25 mM Tris (pH 8.0), 150 mM NaCl). Plates were incubated at 25°C for 1 h then washed 3× in TBST using a plate washer (BioTek). Plates were blocked with 200 µL of 5% non-fat milk in TBST for 1 h at 25°C. Plates were washed 3× in TBST and 1:3 or 1:5 serial dilutions of mouse sera were made in 5% non-fat milk in TBST starting at 1:50 and incubated at 25°C for 1 h. Plates were washed 3× in TBST, then anti-mouse IgG horseradish peroxidase-conjugated antibodies (Cell Signaling Technologies #7076) were diluted 1:2,000 and 100 µL was added to each well and incubated at 25°C for 30 min. Plates were washed 3× in TBST and 100 µL of TMB (SeraCare) was added to every well for 3 min at room temperature. The reaction was quenched with the addition of 100 µL of 1 N HCl. Plates were immediately read at 450 nm on an Epoch2 plate reader. The data was plotted and fit in Prism (GraphPad) using a four parameter logistic curve to determine the reciprocal ED_{50} values. Each plate contained technical duplicates of a positive control to assess assay variability.

Pseudovirus production

The full-length D614G Wuhan-Hu-1 and Omicron BA.2 spike protein-encoding constructs with a 21-amino-acid C-terminal deletion used for pseudovirus assays were previously described *(93–95)*. The full-length Omicron BA.2 construct containing a 21-amino-acid C-terminal deletion was codon optimized, synthesized, and inserted the HDM vector by Genscript. Pseudotyped VSV was produced as previously described *(93–95)*. In brief, HEK293T cells were split into poly-D-lysine-coated 15-cm plates and grown overnight until they reached approximately 70–80% confluency. The cells were washed 3 times with Opti-MEM (Gibco) and transfected with either the D614G Wuhan-Hu-1 or Omicron BA.2 spike protein-encoding constructs using Lipofectamine 2000 (Life Technologies). After 4–6 h, the medium was supplemented with an equal volume of Dulbecco's modified

Eagle medium (DMEM) supplemented with 20% fetal bovine serum (FBS) and 2% penicillin-streptomycin. The cells were incubated for 20–24 h, washed 3 times with DMEM, and infected with VSVΔG-luc. Two hours after VSVΔG-luc infection, the cells were washed an additional five times with DMEM. The cells were grown in DMEM supplemented with anti-VSV-G antibody (I1-mouse hybridoma supernatant diluted 1:25, from CRL-2700, ATCC) for 18–24 h, after which the supernatant was harvested and clarified by low-speed centrifugation at 2,500g for 10 min. The supernatant was then filtered (0.45 μm) and virus stocks were concentrated 10 times using a 30 kDa centrifugal concentrator (Amicon Ultra). The pseudotyped viruses were then aliquoted and frozen at −80 °C.

Pseudovirus neutralization assays

VeroE6 cells expressing transmembrane serine protease (VeroE6-TMPRSS2) *(96)* were split into white-walled, clear-bottom 96-well plates (Corning) and grown overnight until they reached approximately 70% confluency. Plasma was diluted in DMEM and serially diluted in DMEM at a 1:3 dilution thereafter. Pseudotyped VSV was diluted at a 1:20 to 1:100 ratio in DMEM and an equal volume was added to the diluted plasma. The virus–plasma mixture was incubated for 30 min at room temperature and added to the Vero E6-TMPRSS2 cells. After two hours, an equal volume of DMEM supplemented with 20% FBS and 2% penicillin-streptomycin was added to the cells. After 20–24 h, ONE-Glo EX (Promega) was added to each well and the cells were incubated for 5 min at 37 °C. Luminescence values were measured using a BioTek Synergy Neo2 plate reader. Luminescence readings from the neutralization assays were normalized and analyzed using GraphPad Prism 9.4.1. The Relative Light Unit (RLU) values recorded from uninfected cells were used to define 100% neutralization and RLU values recorded from cells infected with pseudovirus without plasma were used to define 0% neutralization. Reciprocal ID_{50} values were determined in Prism (GraphPad) from the normalized data points using a log(inhibitor) versus normalized response–variable slope model to generate the curve fits. Each neutralization experiment was conducted at least twice using independently produced batches of pseudoviruses. Representative data from a single experiment are provided.

Evaluation of T cell responses

Eight- to twelve-week-old female C57BL/6 mice were immunized intramuscularly at weeks 0 and 3 with diluted adjuvanted protein immunogen or diluted mRNA-LNP. Lungs and spleens from immunized animals were harvested at week 6 and lymphocyte isolation from both tissues was performed. Spleens were dissociated into a single cell suspension using a syringe plunger and passed through a 70 μm strainer. Following centrifugation, red blood cell (RBC) lysis was performed by adding 1mL ACK Lysing Buffer (Quality Biological Inc. #118156101CS) and incubating at room temperature for 3 min. The reaction was quenched by adding 10 mL PBS containing 2% FBS (PBS–2% FBS) and cells were counted and plated for stimulation assay. Lungs were dissociated using a GentleMACS dissociator in 5 mL PBS–2% FBS containing Collagenase IV (1 mg/mL) and 20 kU of DNAse I. Following dissociation, lungs were incubated for 40 minutes at 37 °C while shaking. Following incubation, cells were passed through a 70 μm strainer and centrifuged. RBC lysis was then performed as described above.

Lymphocytes from lungs and spleens were plated in a 96-well round-bottom plate at a density of 1×10^6 cells/mL in 200 μL final volume with complete T cell media (containing RPMI-1640, FBS, penicillin-streptomycin, non-essential amino acids, β-mercaptoethanol, and HEPES). Cells were stimulated with an overlapping peptide pool derived from SARS-CoV-2 spike protein (Genscript). Following 2 hours of culture, Brefeldin A (10 μg/mL; eBioscience #00-4506-51) was added, and cells were left in culture for 8 more hours. Following the stimulation, cells were washed with PBS and stained with Ghost Dye Violet 510 fixable viability dye (BV510; Tonbo Biosciences #10140-892, 1:500 dilution), anti-CD3 (BV786; BioLegend #100355, 1:50 dilution), anti-CD4

(BV650; BioLegend #100555; 1:200 dilution), anti-CD8 (BV711; BD #563046; 1:200 dilution), and anti-CD45 (BUV395, BD #564279). Cells were fixed and permeabilized with BD Cytofix/Cytoperm, then stained intracellularly with anti-IFN-γ (APC; BioLegend #505810; 1:100 dilution), anti-TNF-α (FITC; BioLegend #506304, 1:100 dilution), and anti-IL-2 (PE; BioLegend #503808, 1:100 dilution). Cells were analyzed with a BD FACSymphony (5 lasers: UV, Violet, Blue, Yellow, Red; 29 parameters: 7-UV, 8-Violet, 6-Blue, 5-Yellow, and 3-Red) analyzer at the Stanford Shared FACS Facility (SSFF). All flow cytometry data were analyzed using FlowJo software v10.

Viruses

The mouse-adapted viruses (Wuhan-Hu-1 SARS-CoV-2 MA10 and Omicron BA.5 SARS-CoV-2 MA10) and authentic viruses (D614G Wuhan-Hu-1 SARS-CoV-2 and Omicron BA.5 SARS-CoV-2, both expressing nanoLuciferase) used in this study were described previously *(53, 56, 97, 98)*. All virus stocks were propagated in VeroE6-TMPRSS2 cells. Stocks were subjected to next-generation sequencing (Illumina) to confirm the introduction and stability of substitutions.

Authentic virus neutralization assays

Pre-challenge sera were serially diluted (1:20 initially, followed by a 5-fold dilution) in 96-well round-bottom plates (Corning 3799) and incubated at a 1:1 ratio with nanoLuciferase-expressing D614G Wuhan-Hu-1 SARS-CoV-2 or nanoLuciferase-expressing Omicron BA.5 SARS-CoV-2 for 1 hour at 37°C, 5% CO_2. Following incubation, the serum-virus complexes were added, in duplicate, to confluent monolayers of Vero C1008 cells (ATCC, cat# CRL-1586) and incubated for 26-30 hours, depending on the virus. After incubation, RLU values were measured with the Nano-Glo Luciferase Assay System (Promega) according to the manufacturer's protocol. Neutralization titers were calculated as the dilution at which a 50% reduction in RLU values was observed relative to the virus-only control. Curve fits were generated using a macro constrained at the top (100% neutralization) but not the bottom (0% neutralization). Assays were performed once with technical duplicates.

Wuhan-Hu-1 and Omicron BA. 5 SARS-CoV-2 MA10 challenge

Animal studies were carried out in accordance with the Institutional Animal Care and Use Committee at UNC Chapel Hill (protocol number 23-085) and were performed in approved biosafety level 3 (BSL-3) facilities. Ten-week-old female BALB/c mice (Inotiv, code 047; groups of 4-6 mice per group per time point) were vaccinated intramuscularly into the quadriceps muscle of both hind legs with 25 µL of diluted mRNA-LNP or PBS per injection site on week 0 (Wuhan-Hu-1 study) and 4 (Omicron BA.5 study). On week 5 (Wuhan-Hu-1) or 8 (Omicron BA.5 study), serum was collected and mice were moved into BSL-3 for challenge the following week. Briefly, mice were anesthetized with a mixture of ketamine/xylazine, then inoculated intranasally with 1×10^5 PFU of Wuhan-Hu-1 or Omicron BA.5 SARS-CoV-2 MA10. Mice were then monitored daily for clinical signs of disease, weight loss, and mortality. At 2 and 4 days post infection (dpi), mice were euthanized by isoflurane overdose, and the congestion score was assessed (4 dpi only for the Wuhan-Hu-1 study). The inferior lung lobe and nasal turbinates (for 2 dpi only) were collected in PBS with glass beads and stored at -80ºC for viral titer determination by plaque assay, as previously described *(53)*. Briefly, harvested tissues were homogenized in PBS and serial dilutions of the clarified homogenates were added to a confluent monolayer of VeroE6 cells followed by agarose overlay. Plaques were then visualized by staining with Neutral Red dye.

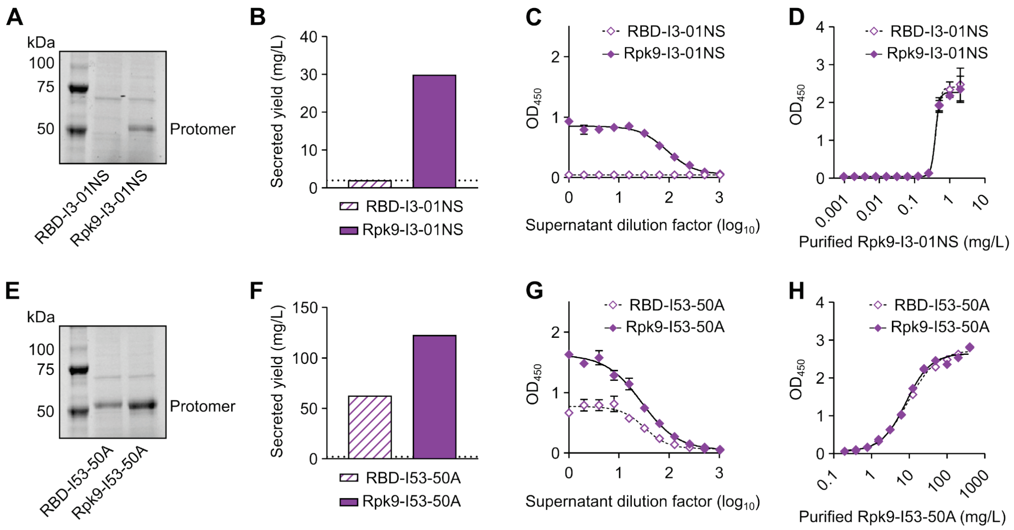

Figure S1. Quantification of secreted severe acute respiratory syndrome coronavirus 2 (SARS-CoV-2) receptor binding domain (RBD) nanoparticles and RBD trimers.

(A) Reducing SDS-PAGE of supernatants from Expi293F cells after expression of RBD nanoparticles. **(B)** Secreted yield of RBD nanoparticles, as determined by cell supernatant enzyme-linked immunosorbent assays (ELISAs). **(C)** RBD nanoparticle supernatant binding against CV30. OD, optical density. **(D)** Purified Rpk9-I3-01NS binding against CV30 was used to quantify the secreted yield of both supernatants. The standard curves generated for analysis are shown for each supernatant. **(E)** Reducing SDS-PAGE of supernatants from Expi293F cells after expression of RBD trimers. **(F)** Secreted yield of RBD trimers, as determined by supernatant ELISAs. **(G)** RBD trimer supernatant binding against CV30. **(H)** Purified Rpk9-I53-50A binding against CV30 was used to quantify the secreted yield of both supernatants. The standard curves generated for analysis are shown for each supernatant. Note the different x axis scales in (D) and (H), highlighting that nanoparticles and trimers require distinct standard curves for accurate quantitation. For all panels**,** representative data are shown from one of three biological replicates for each construct. For (B) and (F), the dotted horizontal line represents the limit of detection for the assay. For (C, D) and (G, H), error bars represent mean ± SD of two technical replicates.

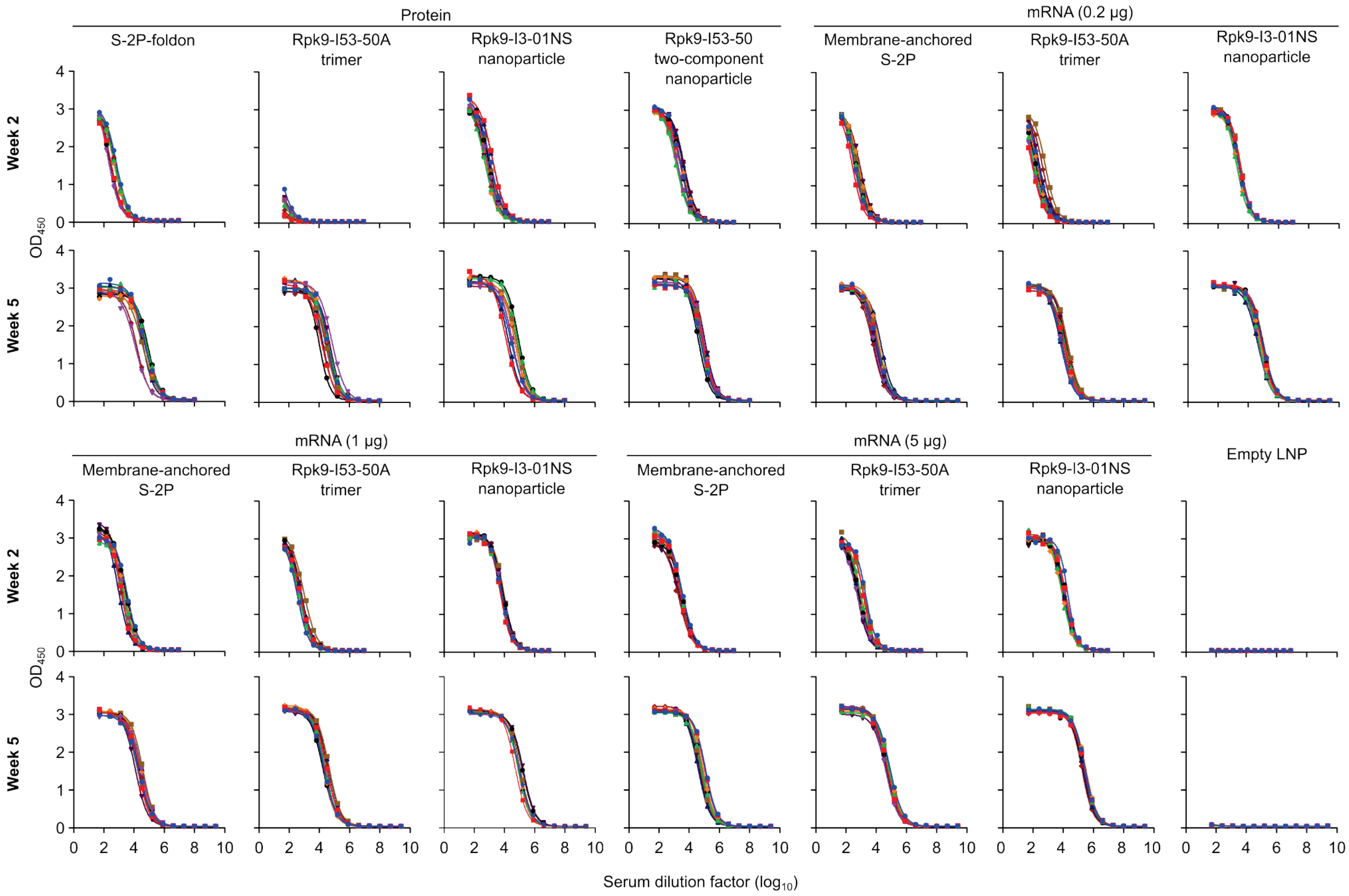

Figure S2. Raw ELISA data and fits used to determine titers shown in Fig. 2.
Serum binding against Wuhan-Hu-1 SARS-CoV-2 HexaPro. Each color represents an individual animal.

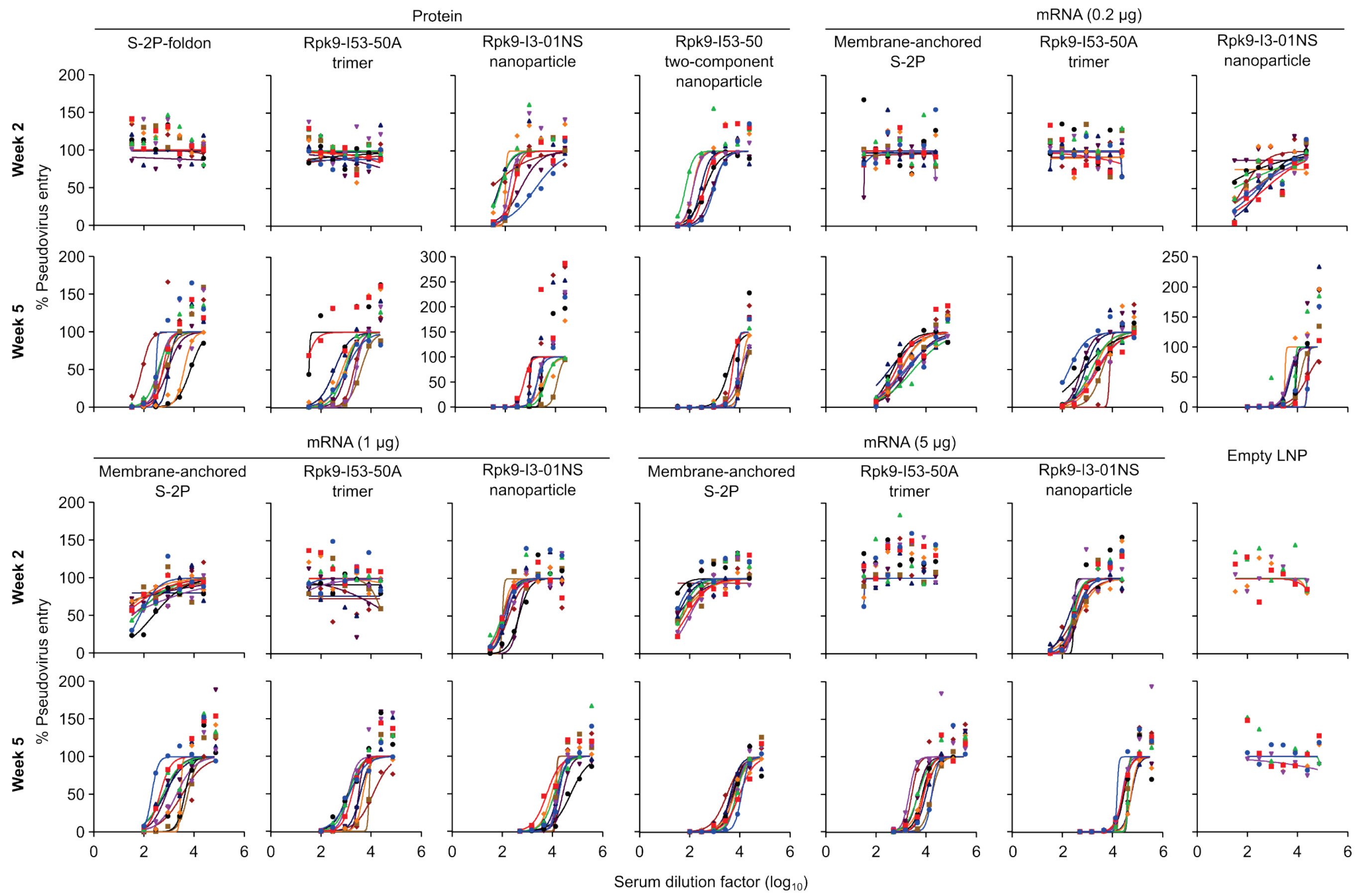

Figure S3. Dose-response curves of D614G Wuhan-Hu-1 SARS-CoV-2 pseudovirus neutralization.
Serum neutralizing activity against VSV pseudotyped with D614G Wuhan-Hu-1 SARS-CoV-2 spike protein. Each color represents an individual animal. Some groups do not have data for all immunized mice due to a lack of available sera (for week 2: n=9 for S-2P-foldon, n=8 for Rpk9-I53-50, n=9 for Rpk9-I3-01NS (protein), and n=4 for Empty LNP). The y axis range and scale of each plot correspond to the leftmost graph in the row unless otherwise indicated.

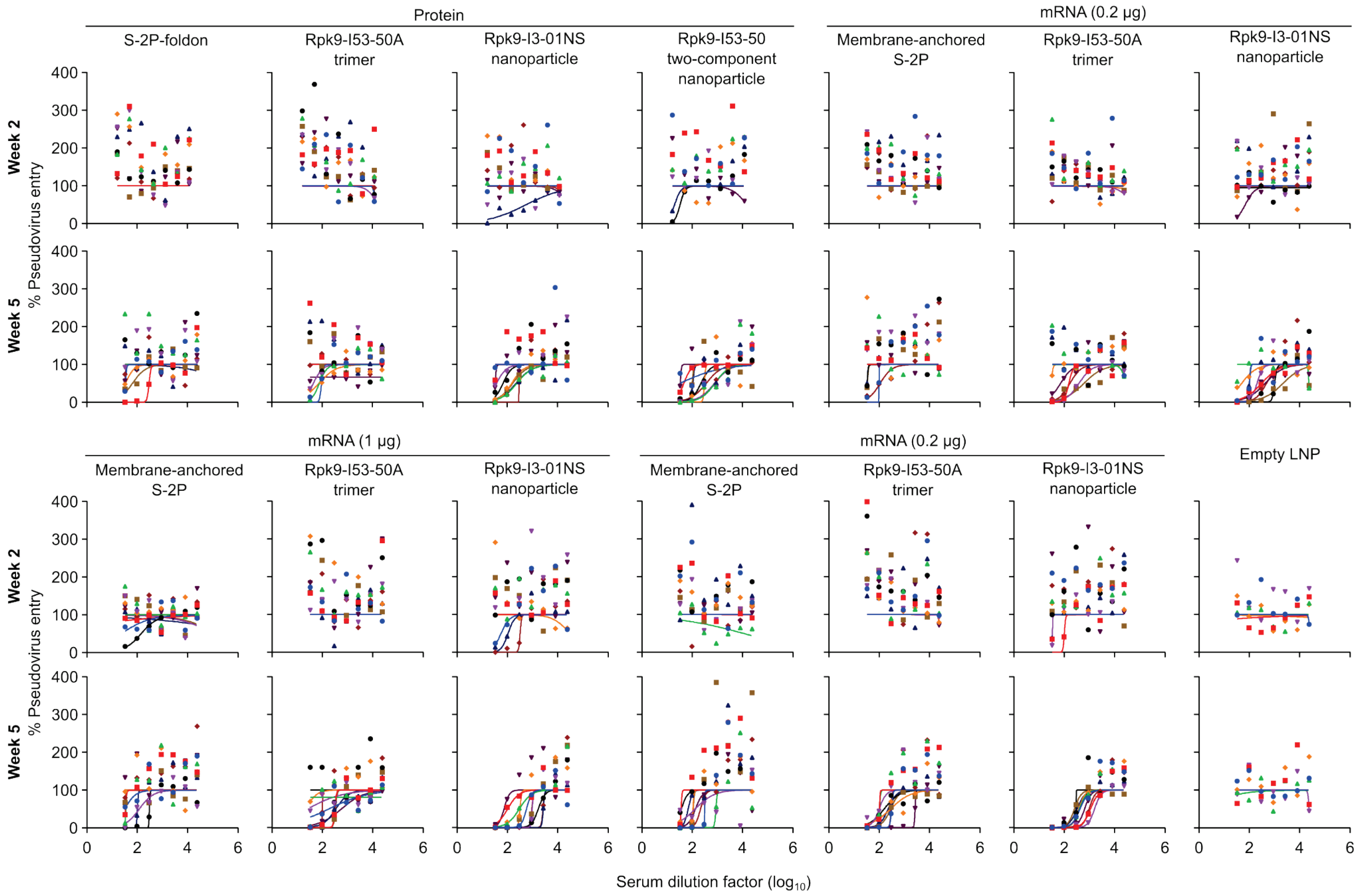

Figure S4. Dose-response curves of Omicron BA.2 SARS-CoV-2 pseudovirus neutralization.
Serum neutralizing activity against VSV pseudotyped with Omicron BA.2 SARS-CoV-2 spike protein. Each color represents an individual animal. Some groups do not have data for all immunized mice due to a lack of available sera (for week 2: n=9 for S-2P-foldon, n=8 for Rpk9-I53-50, n=9 for Rpk9-I3-01NS (protein)).

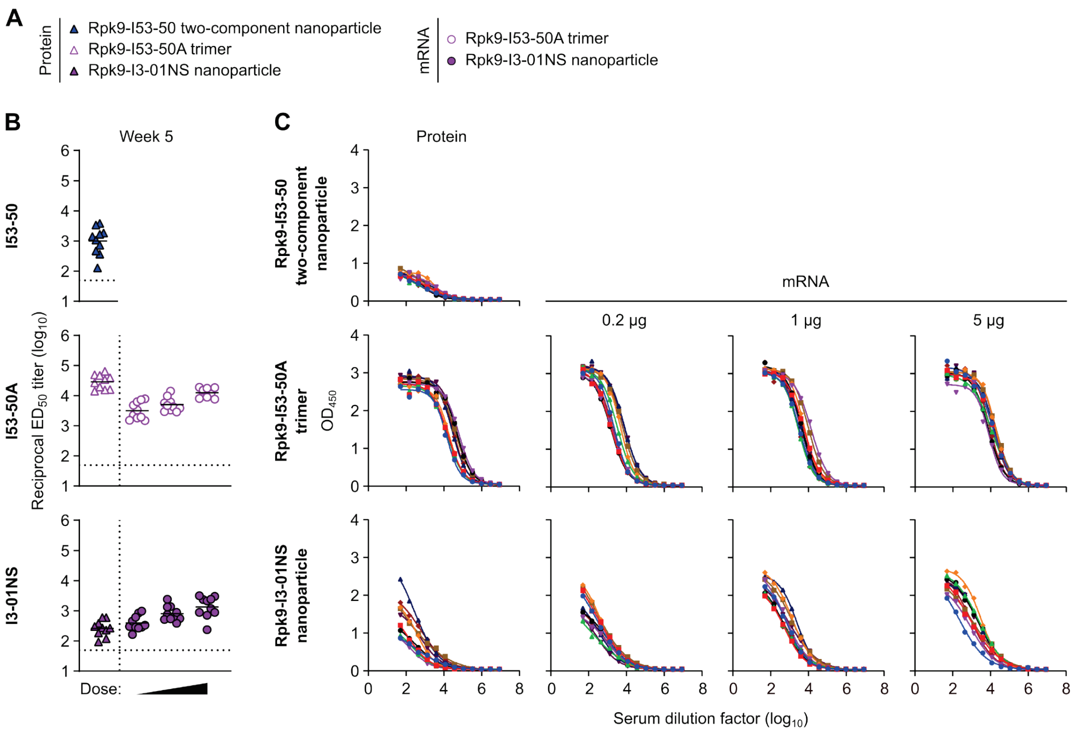

Figure S5. Anti-scaffold antibody titers in BALB/c mice two weeks post-boost.
(A) Groups assessed for anti-scaffold responses at week 5 (from mice in Fig. 2). **(B)** Serum antibody binding titers against the I53-50, I53-50A, and I3-01NS scaffolds. Each symbol represents an individual animal and the geometric mean titer (GMT) from each group is indicated by a horizontal line. The dotted horizontal line represents the limit of detection for the assay. The dotted vertical line separates the protein and mRNA immunized groups. ED_{50}, half-maximal effective dilution. **(C)** Raw ELISA data and fits used to determine titers in (B). Each colored curve represents an individual animal.

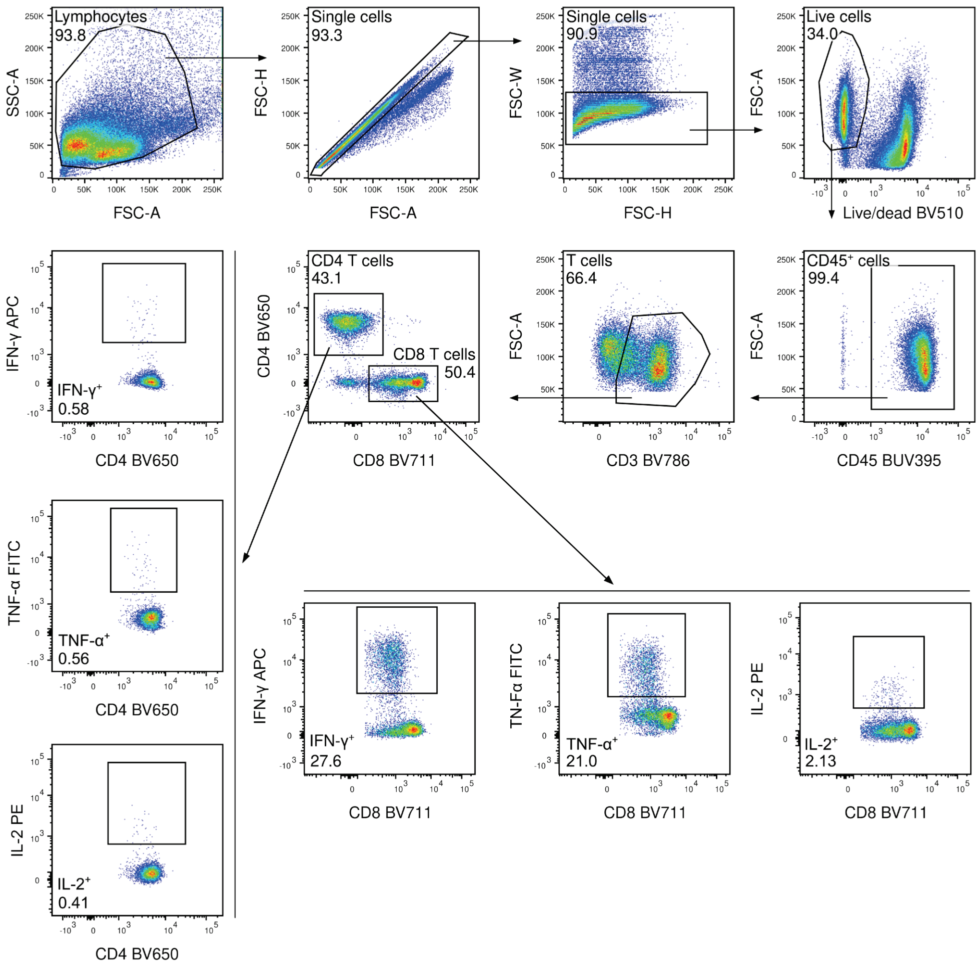

Figure S6. Gating strategy for evaluating antigen-specific CD4 and CD8 T cells.
Representative data are shown from the lungs of a mouse immunized with mRNA encoding for membrane-anchored S-2P. IFN-γ, interferon-γ; TNF-α, tumor necrosis factor-α; IL-2, interleukin-2.

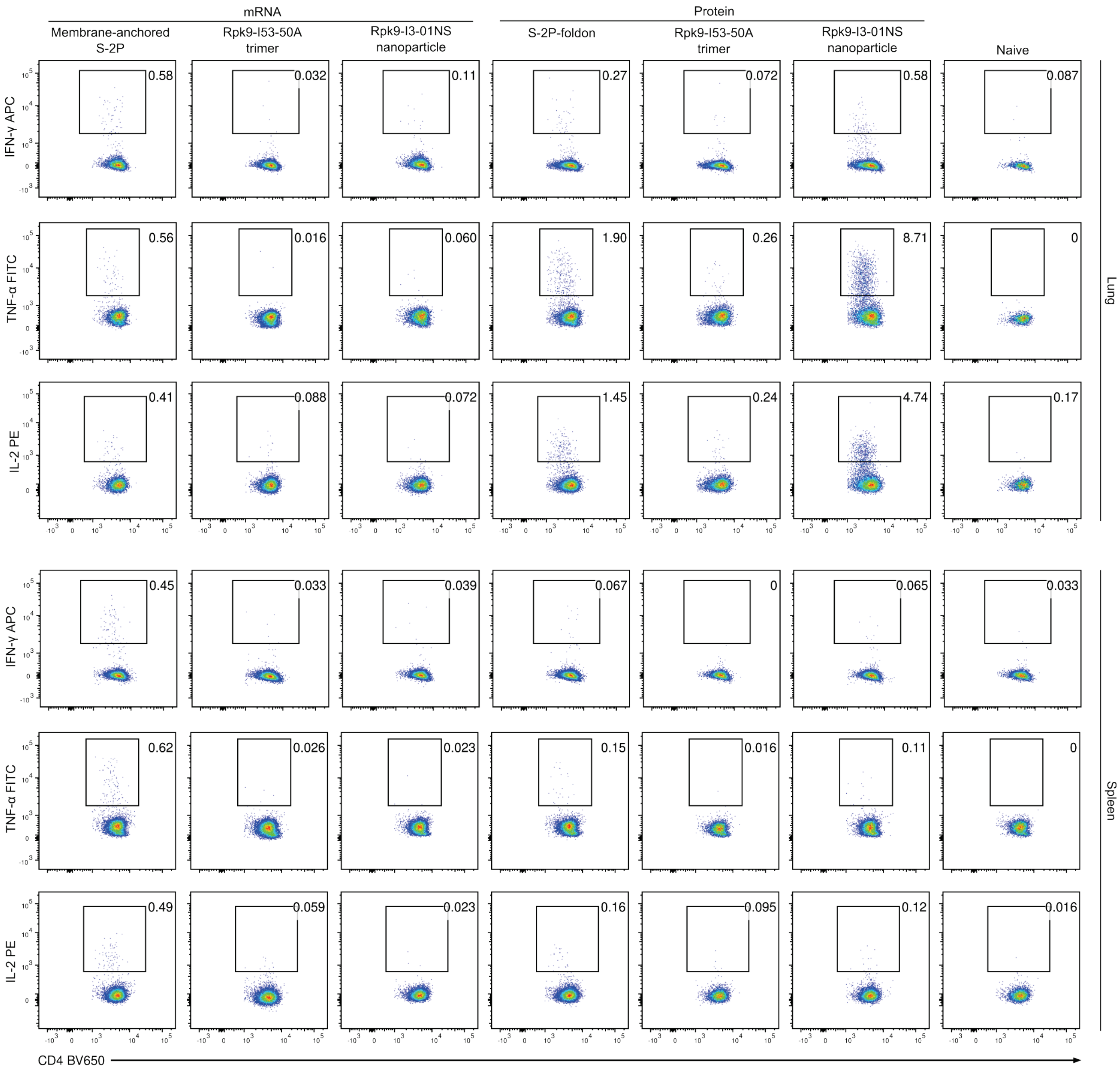

Figure S7. CD4 T cell responses in C57BL/6 mice three weeks post-boost.
Representative flow cytometry plots of CD4 T cell cytokine production for all groups.

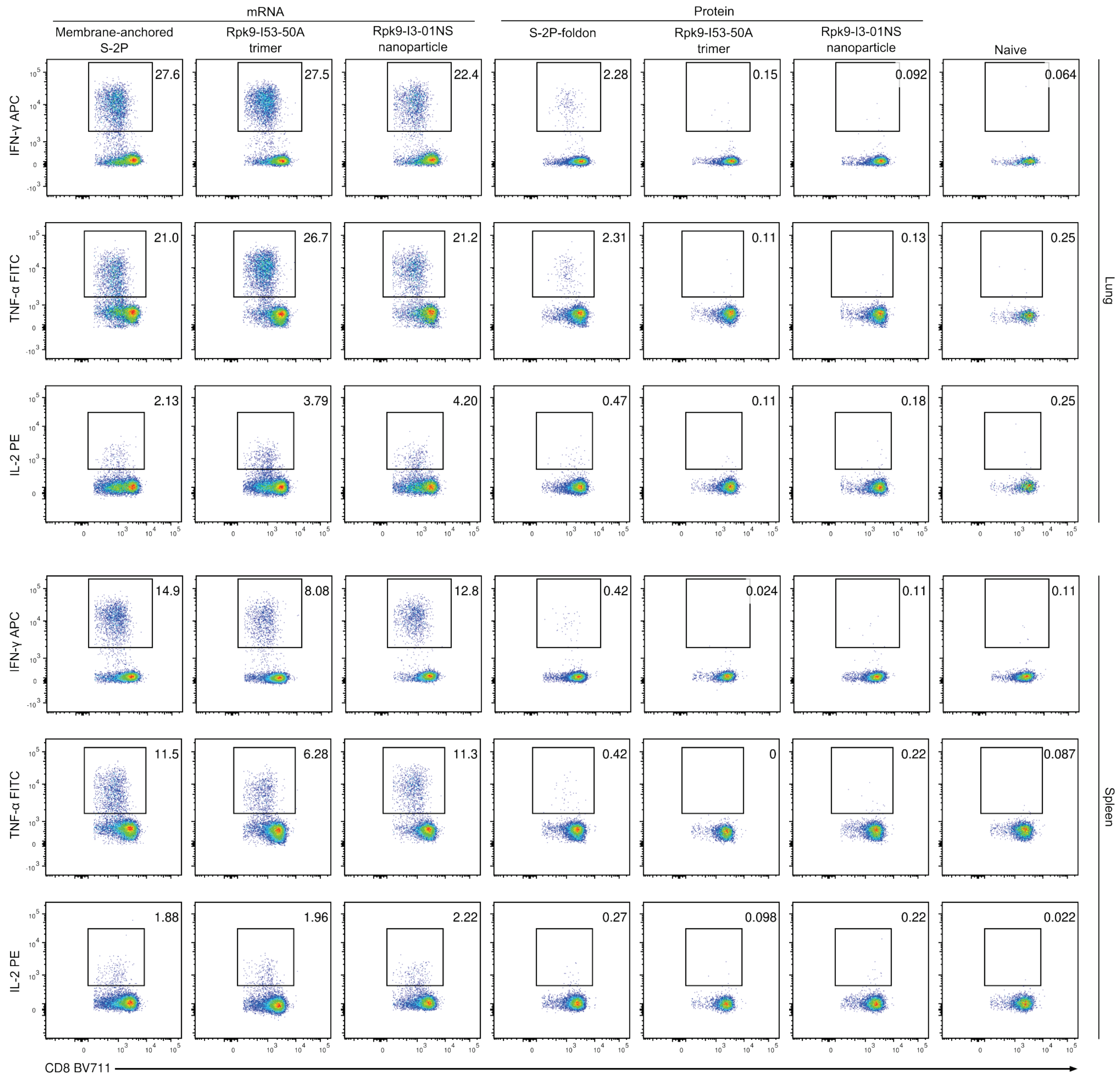

Figure S8. CD8 T cell responses in C57BL/6 mice three weeks post-boost.
Representative flow cytometry plots of CD8 T cell cytokine production for all groups.

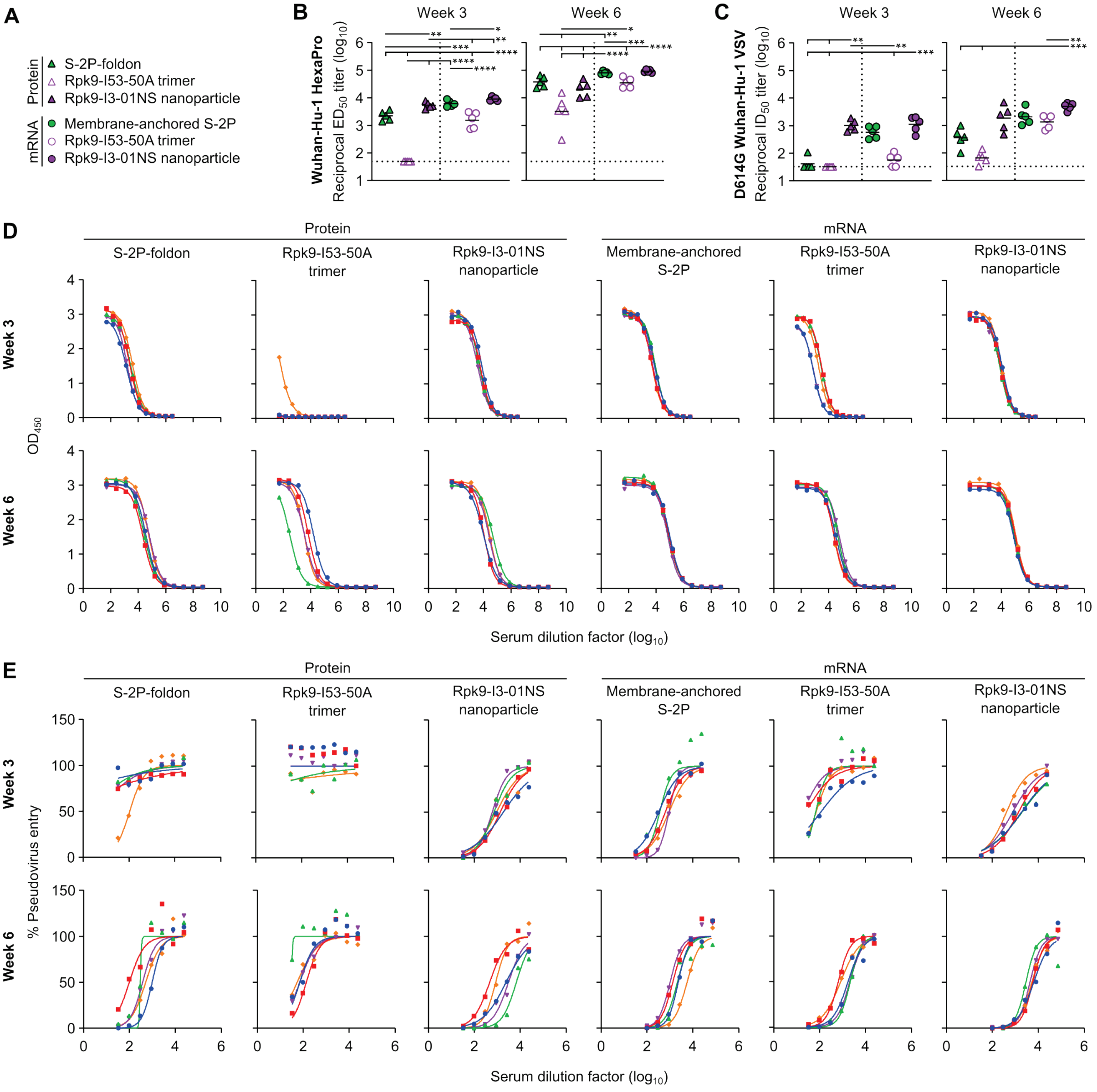

Figure S9. Antigen-specific and neutralizing antibody titers in C57BL/6 mice.

(A) Groups were assessed for antigen-specific and neutralizing antibody responses (from mice in Fig. 3). **(B)** Serum antibody binding titers against Wuhan-Hu-1 SARS-CoV-2 HexaPro were measured by ELISA. **(C)** Serum neutralizing antibody titers against VSV pseudotyped with D614G Wuhan-Hu-1 SARS-CoV-2 spike protein. **(D)** Raw ELISA data and fits used to determine titers in (B). **(E)** Raw D614G Wuhan-Hu-1 SARS-CoV-2 pseudovirus entry data and fits used to determine titers in (C). For (B and C), the GMT from each group is indicated by a horizontal line. The dotted horizontal line represents the lowest limit of detection among the plotted data, as the limits of detection vary between groups. Statistical significance was determined using one-way ANOVA followed by Tukey's multiple comparisons test; $*p < 0.05$, $**p < 0.01$, $***p < 0.001$, and $****p < 0.0001$. For (D and E)**,** each color represents an individual animal.

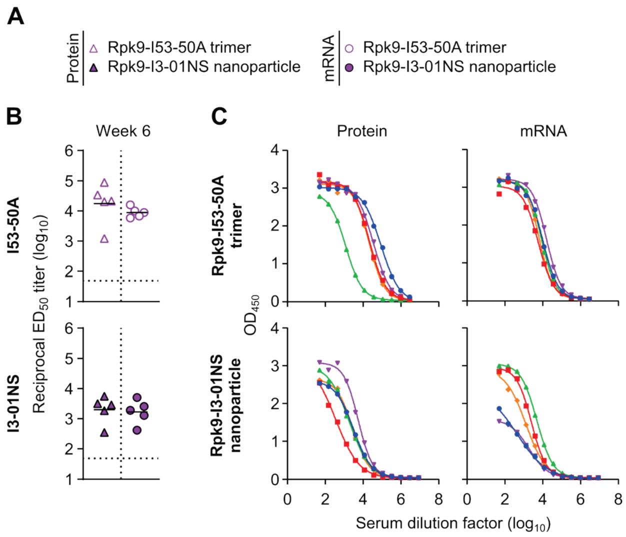

Figure S10. Anti-scaffold antibody titers in C57BL/6 mice three weeks post-boost.
(A) Groups assessed for anti-scaffold responses at week 6 (from mice in Fig. 3). **(B)** Serum antibody binding titers against the I53-50A and I3-01NS scaffolds. Each symbol represents an individual animal and the GMT from each group is indicated by a horizontal line. The dotted horizontal line represents the limit of detection for the assay. **(C)** Raw ELISA data and fits used to determine titers in (B). Each color represents an individual animal.

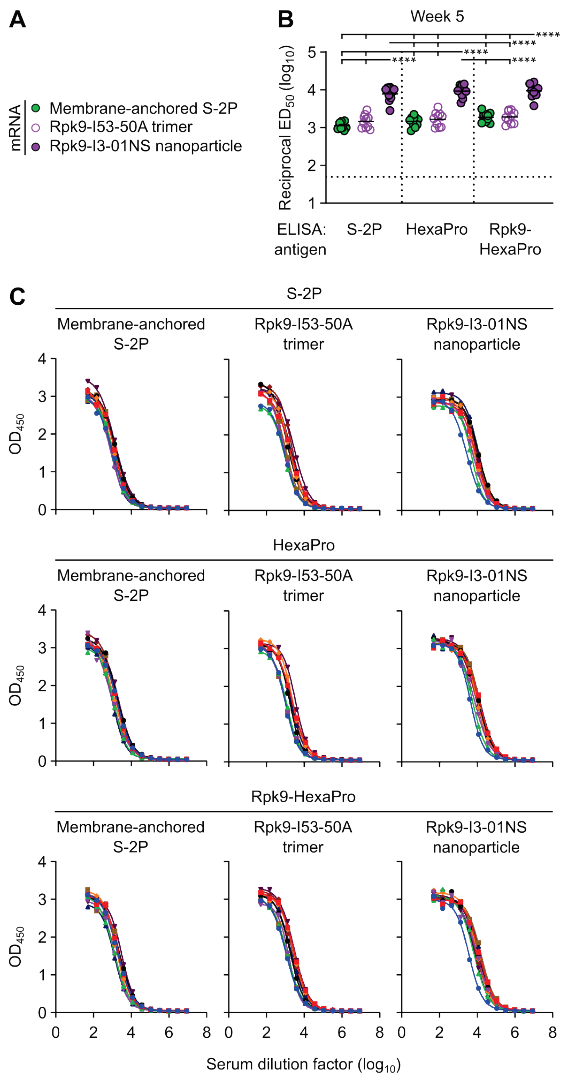

Figure S11. Antigen-specific antibody titers in BALB/c mice five weeks post-immunization.
(A) Groups were assessed for antigen-specific responses at week 5 (from mice in Fig. 4). **(B)** Serum antibody binding titers were measured against three Wuhan-Hu-1 SARS-CoV-2 spike antigens (S-2P, HexaPro, and Rpk9-HexaPro) by ELISA. Each symbol represents an individual animal and the GMT from each group is indicated by a horizontal line. The dotted horizontal line represents the limit of detection for the assay. Statistical significance was determined using one-way ANOVA followed by Tukey's multiple comparisons test; ****$p < 0.0001$. **(C)** Raw ELISA data and fits used to determine titers in (B). Each color represents an individual animal.

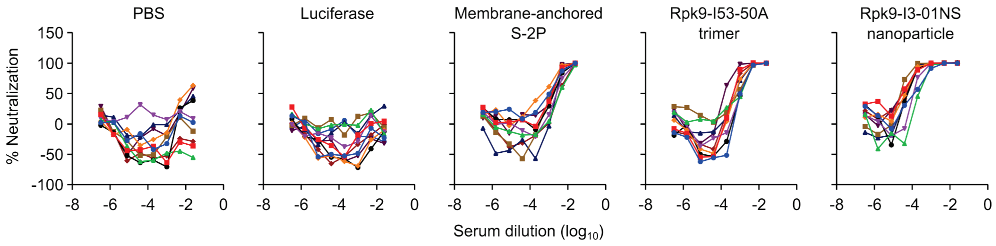

Figure S12. Raw data curves of D614G Wuhan-Hu-1 SARS-CoV-2 authentic virus neutralization data.
Serum neutralizing activity against D614G Wuhan-Hu-1 SARS-CoV-2 authentic virus. Each color represents an individual animal. The mean of two technical replicates are shown.

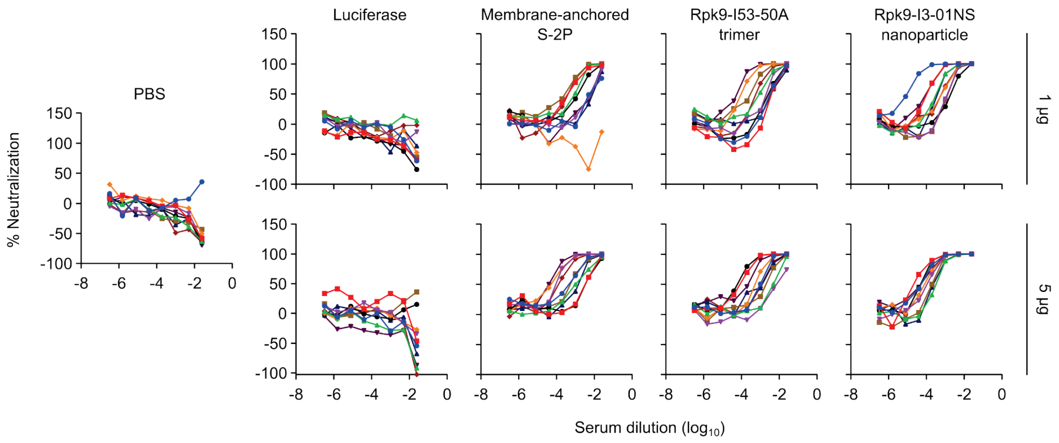

Figure S13. Raw data curves of Omicron BA.5 SARS-CoV-2 authentic virus neutralization data.
Serum neutralizing activity against Omicron BA.5 SARS-CoV-2 authentic virus. Each color represents an individual animal. The mean of two technical replicates are shown.

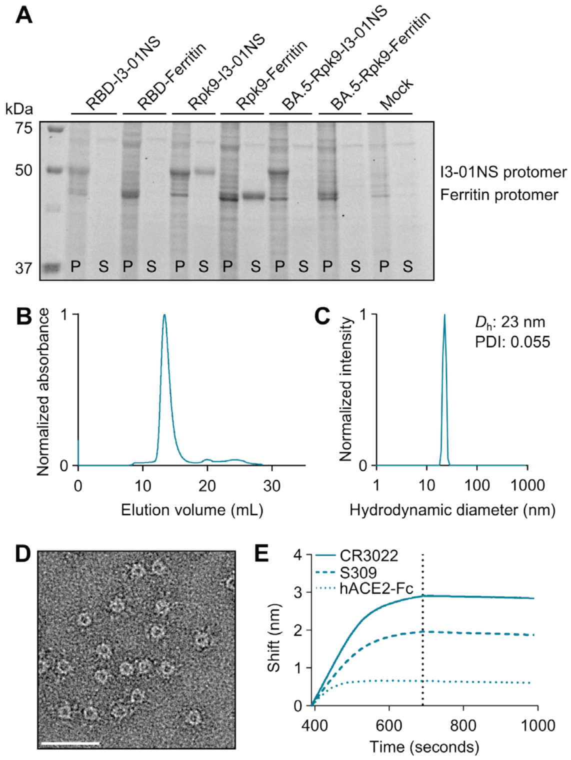

Figure S14. Secretion and characterization of Rpk9-Ferritin.

(A) Reducing SDS-PAGE of Expi293F cell pellets and supernatants after transfection of protein nanoparticle immunogens. Mock transfection included for reference. P, cell pellet; S, supernatant. **(B)** Size exclusion chromatogram of Rpk9-Ferritin purification. **(C)** Dynamic light scattering (DLS) of size exclusion chromatography (SEC)-purified Rpk9-Ferritin. D_h, hydrodynamic diameter; PDI, polydispersity index. **(D)** Representative electron micrograph (scale bar = 25 nm) of negatively stained SEC-purified Rpk9-Ferritin. **(E)** Binding of immobilized Fc-tagged receptor or IgG to SEC-purified Rpk9-Ferritin as assessed by biolayer interferometry (BLI). The dotted vertical line separates the association and dissociation steps.

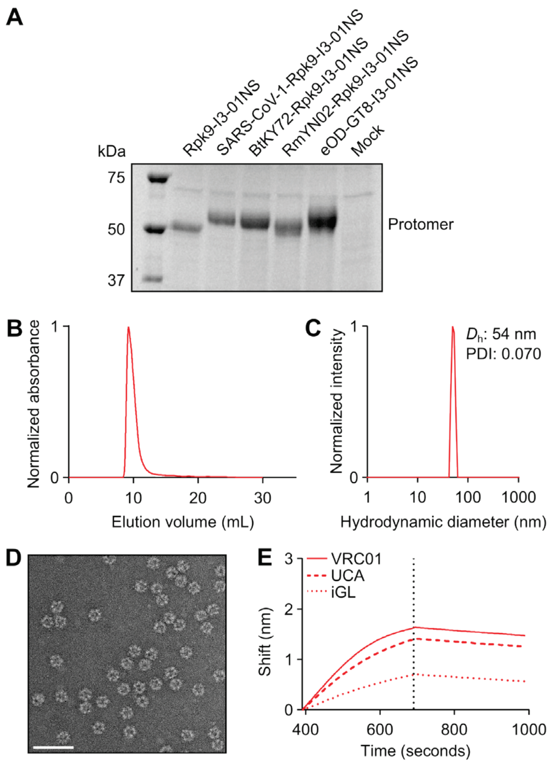

Figure S15. Secretion and characterization of sarbecovirus Rpk9-I3-01NS nanoparticles and eOD-GT8-I3-01NS. **(A)** Reducing SDS-PAGE of supernatants from Expi293F cells after expression of protein nanoparticle immunogens. Mock transfection included for reference. **(B)** Size exclusion chromatogram of eOD-GT8-I3-01NS purification. **(C)** DLS of SEC-purified eOD-GT8-I3-01NS. D_h, hydrodynamic diameter; PDI, polydispersity index. **(D)** Representative electron micrograph (scale bar = 50 nm) of negatively stained SEC-purified eOD-GT8-I3-01NS. **(E)** Binding of immobilized VRC01 IgGs (mature, inferred germline (iGL), and unmutated common ancestor (UCA)) to SEC-purified eOD-GT8-I3-01NS as assessed by BLI. The dotted vertical line separates the association and dissociation steps.

Supplementary Tables

Table S1. Amino acid sequences of proteins described in this study.

**>RBD-I3-01NS**<br>(MGILPSPGMPALLSLVSLLSVLLMGCVAETGT)RFPNITNLCPFGEVFNATRFASVYAWNRKRISNCVADYSVLYNSASF<br>STFKCYGVSPTKLNDLCFTNVYADSFVIRGDEVRQIAPGQTGKIADYNYKLPDDFTGCVIAWNSNNLDSKVGGNYNYLY<br>RLFRKSNLKPFERDISTEIYQAGSTPCNGVEGFNCYFPLQSYGFQPTNGVGYQPYRVVVLSFELLHAPATVCGPKKSTG<br>GSGGSGSGGSGGSGSEKAAKAEEAARKMEELFKEHKIVAVLRANSVEEAKKKALAVFLGGVDLIEITFTVPDADTVIKEL<br>SFLKEMGAIIGAGTVTSVEQAREAVESGAEFIVSPHLDEEISQFAKEEGVFYMPGVMTPTELVKAMKLGHTILKLFPGEVV<br>GPQFVEAMKGPFPNVKFVPTGGVNLDNVAEWFEAGVQAVGVGEALNEGTPVEVAEKAKAFVEKIEGATE
**>Rpk9-I3-01NS**<br>(MGILPSPGMPALLSLVSLLSVLLMGCVAETGT)RFPNITNLCPFGEVFNATRFASVYAWNRKRISNCVADFSVLYNSASF<br>STFKCYGVSPTKLNDLCWTNIYADSFVIRGDEVRQIAPGQTGKIADYNYKLPDDFTGCVIAWNSNNLDSKVGGNYNYLY<br>RLFRKSNLKPFERDISTEIYQAGSTPCNGVEGFNCYFPLQSYGFQPTNGVGYQPYRVVVLSFELLHAPATVCGPKKSTG<br>GSGGSGSGGSGGSGSEKAAKAEEAARKMEELFKEHKIVAVLRANSVEEAKKKALAVFLGGVDLIEITFTVPDADTVIKEL<br>SFLKEMGAIIGAGTVTSVEQAREAVESGAEFIVSPHLDEEISQFAKEEGVFYMPGVMTPTELVKAMKLGHTILKLFPGEVV<br>GPQFVEAMKGPFPNVKFVPTGGVNLDNVAEWFEAGVQAVGVGEALNEGTPVEVAEKAKAFVEKIEGATE
**>RBD-Ferritin**<br>(MGILPSPGMPALLSLVSLLSVLLMGCVAETGT)RFPNITNLCPFGEVFNATRFASVYAWNRKRISNCVADYSVLYNSASF<br>STFKCYGVSPTKLNDLCFTNVYADSFVIRGDEVRQIAPGQTGKIADYNYKLPDDFTGCVIAWNSNNLDSKVGGNYNYLY<br>RLFRKSNLKPFERDISTEIYQAGSTPCNGVEGFNCYFPLQSYGFQPTNGVGYQPYRVVVLSFELLHAPATVCGPKKSTG<br>GSGGSGSGGSGGSGSDIIKLLNEQVNKEMQSSNLYMSMSSWCYTHSLDGAGLFLFDHAAEEYEHAKKLIIFLNENNVPV<br>QLTSISAPEHKFEGLTQIFQKAYEHEQHISESINNIVDHAIKSKDHATFNFLQWYVAEQHEEEVLFKDILDKIELIGNENHGL<br>YLADQYVKGIAKSRKS
**>Rpk9-Ferritin**<br>(MGILPSPGMPALLSLVSLLSVLLMGCVAETGT)RFPNITNLCPFGEVFNATRFASVYAWNRKRISNCVADFSVLYNSASF<br>STFKCYGVSPTKLNDLCWTNIYADSFVIRGDEVRQIAPGQTGKIADYNYKLPDDFTGCVIAWNSNNLDSKVGGNYNYLY<br>RLFRKSNLKPFERDISTEIYQAGSTPCNGVEGFNCYFPLQSYGFQPTNGVGYQPYRVVVLSFELLHAPATVCGPKKSTG<br>GSGGSGSGGSGGSGSDIIKLLNEQVNKEMQSSNLYMSMSSWCYTHSLDGAGLFLFDHAAEEYEHAKKLIIFLNENNVPV<br>QLTSISAPEHKFEGLTQIFQKAYEHEQHISESINNIVDHAIKSKDHATFNFLQWYVAEQHEEEVLFKDILDKIELIGNENHGL<br>YLADQYVKGIAKSRKS
**>BA.5-Rpk9-I3-01NS**<br>(MGILPSPGMPALLSLVSLLSVLLMGCVAETGT)RFPNITNLCPFDEVFNATRFASVYAWNRKRISNCVADFSVLYNFAPF<br>FAFKCYGVSPTKLNDLCWTNIYADSFVIRGNEVSQIAPGQTGNIADYNYKLPDDFTGCVIAWNSNKLDSKVGGNYNYRY<br>RLFRKSNLKPFERDISTEIYQAGNKPCNGVAGVNCYFPLQSYGFRPTYGVGHQPYRVVVLSFELLHAPATVCGPKKSTG<br>GSGGSGSGGSGGSGSEKAAKAEEAARKMEELFKEHKIVAVLRANSVEEAKKKALAVFLGGVDLIEITFTVPDADTVIKEL<br>SFLKEMGAIIGAGTVTSVEQAREAVESGAEFIVSPHLDEEISQFAKEEGVFYMPGVMTPTELVKAMKLGHTILKLFPGEVV<br>GPQFVEAMKGPFPNVKFVPTGGVNLDNVAEWFEAGVQAVGVGEALNEGTPVEVAEKAKAFVEKIEGATE
**>BA.5-Rpk9-Ferritin**<br>(MGILPSPGMPALLSLVSLLSVLLMGCVAETGT)RFPNITNLCPFDEVFNATRFASVYAWNRKRISNCVADFSVLYNFAPF<br>FAFKCYGVSPTKLNDLCWTNIYADSFVIRGNEVSQIAPGQTGNIADYNYKLPDDFTGCVIAWNSNKLDSKVGGNYNYRY<br>RLFRKSNLKPFERDISTEIYQAGNKPCNGVAGVNCYFPLQSYGFRPTYGVGHQPYRVVVLSFELLHAPATVCGPKKSTG<br>GSGGSGSGGSGGSGSDIIKLLNEQVNKEMQSSNLYMSMSSWCYTHSLDGAGLFLFDHAAEEYEHAKKLIIFLNENNVPV<br>QLTSISAPEHKFEGLTQIFQKAYEHEQHISESINNIVDHAIKSKDHATFNFLQWYVAEQHEEEVLFKDILDKIELIGNENHGL<br>YLADQYVKGIAKSRKS

>SARS-CoV-1-Rpk9-I3-01NS
(MGILPSPGMPALLSLVSLLSVLLMGCVAETGT)RFPNITNLCPFGEVFNATKFPSVYAWERKKISNCVADFSVLYNSTFF
STFKCYGVSATKLNDLCWSNIYADSFVVKGDDVRQIAPGQTGVIADYNYKLPDDFMGCVLAWNTRNIDATSTGNYNYKY
RYLRHGKLRPFERDISNVPFSPDGKPCTPPALNCYWPLNDYGFYTTTGIGYQPYRVVVLSFELLNAPATVCGPKLSTGG
SGGSGSGGSGGSGSEKAAKAEEAARKMEELFKEHKIVAVLRANSVEEAKKKALAVFLGGVDLIEITFTVPDADTVIKELS
FLKEMGAIIGAGTVTSVEQAREAVESGAEFIVSPHLDEEISQFAKEEGVFYMPGVMTPTELVKAMKLGHTILKLFPGEVVG
PQFVEAMKGPFPNVKFVPTGGVNLDNVAEWFEAGVQAVGVGEALNEGTPVEVAEKAKAFVEKIEGATE

>BtKY72-Rpk9-I3-01NS
(MGILPSPGMPALLSLVSLLSVLLMGCVAETGT)RFPNITNLCPFGQVFNASNFPSVYAWERLRISDCVADFAVLYNSSSS
FSTFKCYGVSPTKLNDLCWSSIYADYFVVKGDDVRQIAPAQTGVIADYNYKLPDDFTGCVLAWNTNSVDSKSGNNFYYR
LFRHGKIKPYERDISNVLYNSAGGTCSSISQLGCYEPLKSYGFTPTVGVGYQPYRVVVLSFELLNAPATVCGPKKSTGGS
GGSGSGGSGGSGSEKAAKAEEAARKMEELFKEHKIVAVLRANSVEEAKKKALAVFLGGVDLIEITFTVPDADTVIKELSFL
KEMGAIIGAGTVTSVEQAREAVESGAEFIVSPHLDEEISQFAKEEGVFYMPGVMTPTELVKAMKLGHTILKLFPGEVVGP
QFVEAMKGPFPNVKFVPTGGVNLDNVAEWFEAGVQAVGVGEALNEGTPVEVAEKAKAFVEKIEGATE

>RmYN02-Rpk9-I3-01NS
(MGILPSPGMPALLSLVSLLSVLLMGCVAETGT)RFPNITNFCPFDKVFNATRFPNVYAWQRTKISDCIADFTVLYNSTSFS
TFKCYGVSPSKLIDLCWTSIYADTFLIRFSEVRQIAPGETGVIADYNYKLPDDFTGCVLAWNTAQQDIGSYFYRSHRAVKL
KPFERDLSSDENGVRTLSTYDFNPNVPLDYQATRVVVLSFELLNAPATVCGPKLSTGGSGGSGSGGSGGSGSEKAAKA
EEAARKMEELFKEHKIVAVLRANSVEEAKKKALAVFLGGVDLIEITFTVPDADTVIKELSFLKEMGAIIGAGTVTSVEQARE
AVESGAEFIVSPHLDEEISQFAKEEGVFYMPGVMTPTELVKAMKLGHTILKLFPGEVVGPQFVEAMKGPFPNVKFVPTG
GVNLDNVAEWFEAGVQAVGVGEALNEGTPVEVAEKAKAFVEKIEGATE

>eOD-GT8-I3-01NS
(MDSKGSSQKGSRLLLLLVVSNLLLPQGVLA)DTITLPCRPAPPPHCSSNITGLILTRQGGYSNDNTVIFRPSGGDWRDIA
RCQIAGTVVSTQLFLNGSLAEEEVVIRSEDWRDNAKSICVQLNTSVEINCTGAGHCNISRAKWNNTLKQIASKLREQYGN
KTIIFKPSSGGDPEFVNHSFNCGGEFFYCDSTQLFNSTWFNSTGGSGGSGSGGSGGSGSEKAAKAEEAARKMEELFK
EHKIVAVLRANSVEEAKKKALAVFLGGVDLIEITFTVPDADTVIKELSFLKEMGAIIGAGTVTSVEQAREAVESGAEFIVSPH
LDEEISQFAKEEGVFYMPGVMTPTELVKAMKLGHTILKLFPGEVVGPQFVEAMKGPFPNVKFVPTGGVNLDNVAEWFE
AGVQAVGVGEALNEGTPVEVAEKAKAFVEKIEGATE

Signal peptides are indicated in parentheses.

Table S2. Amino acid sequences of proteins used in immunizations.

**>Rpk9-I3-01NS (identical to the Rpk9-I3-01NS sequence in table S1)**<br>(MGILPSPGMPALLSLVSLLSVLLMGCVAETGT)RFPNITNLCPFGEVFNATRFASVYAWNRKRISNCVADFSVLYNSASF<br>STFKCYGVSPTKLNDLCWTNIYADSFVIRGDEVRQIAPGQTGKIADYNYKLPDDFTGCVIAWNSNNLDSKVGGNYNYLY<br>RLFRKSNLKPFERDISTEIYQAGSTPCNGVEGFNCYFPLQSYGFQPTNGVGYQPYRVVVLSFELLHAPATVCGPKKSTG<br>GSGGSGSGGSGGSGSEKAAKAEEAARKMEELFKEHKIVAVLRANSVEEAKKKALAVFLGGVDLIEITFTVPDADTVIKEL<br>SFLKEMGAIIGAGTVTSVEQAREAVESGAEFIVSPHLDEEISQFAKEEGVFYMPGVMTPTELVKAMKLGHTILKLFPGEVV<br>GPQFVEAMKGPFPNVKFVPTGGVNLDNVAEWFEAGVQAVGVGEALNEGTPVEVAEKAKAFVEKIEGATE
**>Rpk9-I53-50A**<br>(MGILPSPGMPALLSLVSLLSVLLMGCVAETGT)RFPNITNLCPFGEVFNATRFASVYAWNRKRISNCVADFSVLYNSASF<br>STFKCYGVSPTKLNDLCWTNIYADSFVIRGDEVRQIAPGQTGKIADYNYKLPDDFTGCVIAWNSNNLDSKVGGNYNYLY<br>RLFRKSNLKPFERDISTEIYQAGSTPCNGVEGFNCYFPLQSYGFQPTNGVGYQPYRVVVLSFELLHAPATVCGPKKSTG<br>GSGGSGSGGSGGSGSEKAAKAEEAARKMEELFKKHKIVAVLRANSVEEAIEKAVAVFAGGVHLIEITFTVPDADTVIKAL<br>SVLKEKGAIIGAGTVTSVEQARKAVESGAEFIVSPHLDEEISQFAKEKGVFYMPGVMTPTELVKAMKLGHTILKLFPGEVV<br>GPQFVKAMKGPFPNVKFVPTGGVNLDNVAEWFKAGVLAVGVGSALVKGTPDEVREKAKAFVEKIRGATEGGSHHHHH<br>HHH
**>S-2P-foldon**<br>(MGILPSPGMPALLSLVSLLSVLLMGCVAETGT)QCVNLTTRTQLPPAYTNSFTRGVYYPDKVFRSSVLHSTQDLFLPFFS<br>NVTWFHAIHVSGTNGTKRFDNPVLPFNDGVYFASTEKSNIIRGWIFGTTLDSKTQSLLIVNNATNVVIKVCEFQFCNDPFL<br>GVYYHKNNKSWMESEFRVYSSANNCTFEYVSQPFLMDLEGKQGNFKNLREFVFKNIDGYFKIYSKHTPINLVRDLPQGF<br>SALEPLVDLPIGINITRFQTLLALHRSYLTPGDSSSGWTAGAAAYYVGYLQPRTFLLKYNENGTITDAVDCALDPLSETKC<br>TLKSFTVEKGIYQTSNFRVQPTESIVRFPNITNLCPFGEVFNATRFASVYAWNRKRISNCVADYSVLYNSASFSTFKCYG<br>VSPTKLNDLCFTNVYADSFVIRGDEVRQIAPGQTGKIADYNYKLPDDFTGCVIAWNSNNLDSKVGGNYNYLYRLFRKSNL<br>KPFERDISTEIYQAGSTPCNGVEGFNCYFPLQSYGFQPTNGVGYQPYRVVVLSFELLHAPATVCGPKKSTNLVKNKCVN<br>FNFNGLTGTGVLTESNKKFLPFQQFGRDIADTTDAVRDPQTLEILDITPCSFGGVSVITPGTNTSNQVAVLYQDVNCTEV<br>PVAIHADQLTPTWRVYSTGSNVFQTRAGCLIGAEHVNNSYECDIPIGAGICASYQTQTNSPSGAGSVASQSIIAYTMSLG<br>AENSVAYSNNSIAIPTNFTISVTTEILPVSMTKTSVDCTMYICGDSTECSNLLLQYGSFCTQLNRALTGIAVEQDKNTQEVF<br>AQVKQIYKTPPIKDFGGFNFSQILPDPSKPSKRSFIEDLLFNKVTLADAGFIKQYGDCLGDIAARDLICAQKFNGLTVLPPLL<br>TDEMIAQYTSALLAGTITSGWTFGAGAALQIPFAMQMAYRFNGIGVTQNVLYENQKLIANQFNSAIGKIQDSLSSTASALG<br>KLQDVVNQNAQALNTLVKQLSSNFGAISSVLNDILSRLDPPEAEVQIDRLITGRLQSLQTYVTQQLIRAAEIRASANLAATK<br>MSECVLGQSKRVDFCGKGYHLMSFPQSAPHGVVFLHVTYVPAQEKNFTTAPAICHDGKAHFPREGVFVSNGTHWFVT<br>QRNFYEPQIITTDNTFVSGNCDVVIGIVNNTVYDPLQPELDSFKEELDKYFKNHTSPDVDLGDISGINASVVNIQKEIDRLN<br>EVAKNLNESLIDLQELGKYEQYIKGSGRENLYFQGGGGSGYIPEAPRDGQAYVRKDGEWVLLSTFLGHHHHHHHH
**>Rpk9-I53-50 (A component : B component)**<br>(MGILPSPGMPALLSLVSLLSVLLMGCVAETGT)RFPNITNLCPFGEVFNATRFASVYAWNRKRISNCVADFSVLYNSASF<br>STFKCYGVSPTKLNDLCWTNIYADSFVIRGDEVRQIAPGQTGKIADYNYKLPDDFTGCVIAWNSNNLDSKVGGNYNYLY<br>RLFRKSNLKPFERDISTEIYQAGSTPCNGVEGFNCYFPLQSYGFQPTNGVGYQPYRVVVLSFELLHAPATVCGPKKSTG<br>GSGGSGSGGSGGSGSEKAAKAEEAARKMEELFKKHKIVAVLRANSVEEAIEKAVAVFAGGVHLIEITFTVPDADTVIKAL<br>SVLKEKGAIIGAGTVTSVEQARKAVESGAEFIVSPHLDEEISQFAKEKGVFYMPGVMTPTELVKAMKLGHTILKLFPGEVV<br>GPQFVKAMKGPFPNVKFVPTGGVNLDNVAEWFKAGVLAVGVGSALVKGTPDEVREKAKAFVEKIRGATEGGSHHHHH<br>HHH :<br>MNQHSHKDHETVRIAVVRARWHAEIVDACVSAFEAAMRDIGGDRFAVDVFDVPGAYEIPLHARTLAETGRYGAVLGTAF<br>VVNGGIYRHEFVASAVINGMMNVQLNTGVPVLSAVLTPHNYDKSKAHTLLFLALFAVKGMEAARACVEILAAREKIAAGS<br>LEHHHHHH

Signal peptides are indicated in parentheses.

Table S3. Nucleic acid sequences of open reading frames (ORFs) of mRNA constructs used in immunizations.

**>Rpk9-I3-01NS**<br>AΨGGGCAΨCCΨGCCAAGCCCΨGGAAΨGCCΨGCCCΨGCΨGAGCCΨGGΨGΨCCCΨGCΨGΨCΨGΨGCΨGCΨG<br>AΨGGGAΨGCGΨGGCAGAGACCGGCACAAGGΨΨCCCΨAACAΨCACCAAΨCΨGΨGCCCAΨΨCGGCGAGGΨGΨ<br>ΨΨAACGCCACACGCΨΨΨGCCΨCCGΨGΨAΨGCCΨGGAACCGGAAGAGAAΨCΨCΨAAΨΨGCGΨGGCCGACΨΨ<br>CAGCGΨGCΨGΨACAAΨAGCGCCΨCCΨΨCΨCΨACCΨΨΨAAGΨGCΨAΨGGCGΨGAGCCCCACCAAGCΨGAAC<br>GAΨCΨGΨGCΨGGACAAACAΨΨΨACGCCGACΨCCΨΨΨGΨGAΨCCGGGGCGAΨGAAGΨGAGACAGAΨCGCAC<br>CAGGACAGACCGGAAAGAΨCGCAGACΨACAACΨAΨAAGCΨGCCΨGACGAΨΨΨCACAGGCΨGCGΨGAΨCGCC<br>ΨGGAAΨAGCAACAAΨCΨGGAΨΨCCAAAGΨGGGCGGCAACΨACAAΨΨAΨCΨGΨACAGGCΨGΨΨCCGCAAGΨC<br>CAACCΨGAAGCCAΨΨΨGAGCGGGACAΨCΨCCACCGAGAΨCΨACCAGGCCGGCΨCΨACACCCΨGCAACGGCG<br>ΨGGAGGGCΨΨCAAΨΨGΨΨAΨΨΨΨCCCCΨGCAGΨCΨΨACGGCΨΨCCAGCCΨACAAAΨGGCGΨGGGCΨAΨC<br>AGCCAΨACCGGGΨGGΨGGΨGCΨGΨCΨΨΨΨGAGCΨGCΨGCACGCACCAGCAACCGΨGΨGCGGCCCCAAGAA<br>GAGCACAGGAGGCΨCΨGGAGGCAGCGGCΨCCGGAGGCΨCΨGGAGGCAGCGGCΨCCGAGAAGGCCGCCAAGG<br>CCGAGGAGGCCGCCAGGAAGAΨGGAGGAGCΨCΨΨΨAAGGAGCACAAAAΨCGΨGGCGGΨGCΨCAGGGCAAAΨ<br>AGCGΨAGAAGAGGCCAAAAAGAAGGCACΨCGCCGΨCΨΨΨCΨCGGAGGCGΨGGACCΨGAΨAGAGAΨCACAΨΨ<br>CACGGΨGCCAGAΨGCΨGAΨACGGΨCAΨΨAAGGAGCΨGAGΨΨΨΨCΨCAAAGAGAΨGGGCGCCAΨCAΨAGGC<br>GCCGGCACCGΨGACAΨCCGΨGGAGCAGGCCAGAGAAGCCGΨCGAGΨCCGGAGCCGAAΨΨCAΨAGΨGΨCCCC<br>ΨCAΨCΨCGAΨGAGGAGAΨCAGΨCAGΨΨΨGCCAAGGAGGAGGGAGΨGΨΨΨΨAΨAΨGCCCGGAGΨGAΨGACG<br>CCGACGGAGCΨAGΨGAAGGCCAΨGAAACΨGGGCCACACCAΨCCΨCAAGCΨGΨΨΨCCΨGGAGAAGΨGGΨΨG<br>GΨCCCCAAΨΨΨGΨCGAAGCCAΨGAAGGGACCAΨΨΨCCAAACGΨGAAAΨΨCGΨGCCGACCGGAGGAGΨΨAAC<br>ΨΨAGACAACGΨΨGCCGAGΨGGΨΨΨGAGGCAGGCGΨΨCAGGCGGΨCGGGGΨCGGAGAGGCCCΨCAACGAAG<br>GAACACCAGΨGGAAGΨCGCCGAGAAGGCCAAAGCAΨΨΨGΨGGAGAAAAΨCGAGGGAGCCACΨGAAΨGAΨGA
**>Rpk9-I53-50A**<br>AΨGGGCAΨCCΨGCCAAGCCCΨGGAAΨGCCΨGCCCΨGCΨGAGCCΨGGΨGΨCCCΨGCΨGΨCΨGΨGCΨGCΨG<br>AΨGGGAΨGCGΨGGCAGAGACCGGCACAAGGΨΨCCCΨAACAΨCACCAAΨCΨGΨGCCCAΨΨCGGCGAGGΨGΨ<br>ΨΨAACGCCACACGCΨΨΨGCCΨCCGΨGΨAΨGCCΨGGAACCGGAAGAGAAΨCΨCΨAAΨΨGCGΨGGCCGACΨΨ<br>CAGCGΨGCΨGΨACAAΨAGCGCCΨCCΨΨCΨCΨACCΨΨΨAAGΨGCΨAΨGGCGΨGAGCCCCACCAAGCΨGAAC<br>GAΨCΨGΨGCΨGGACAAACAΨΨΨACGCCGACΨCCΨΨΨGΨGAΨCCGGGGCGAΨGAAGΨGAGACAGAΨCGCAC<br>CAGGACAGACCGGAAAGAΨCGCAGACΨACAACΨAΨAAGCΨGCCΨGACGAΨΨΨCACAGGCΨGCGΨGAΨCGCC<br>ΨGGAAΨAGCAACAAΨCΨGGAΨΨCCAAAGΨGGGCGGCAACΨACAAΨΨAΨCΨGΨACAGGCΨGΨΨCCGCAAGΨC<br>CAACCΨGAAGCCAΨΨΨGAGCGGGACAΨCΨCCACCGAGAΨCΨACCAGGCCGGCΨCΨACACCCΨGCAACGGCG<br>ΨGGAGGGCΨΨCAAΨΨGΨΨAΨΨΨΨCCCCΨGCAGΨCΨΨACGGCΨΨCCAGCCΨACAAAΨGGCGΨGGGCΨAΨC<br>AGCCAΨACCGGGΨGGΨGGΨGCΨGΨCΨΨΨΨGAGCΨGCΨGCACGCACCAGCAACCGΨGΨGCGGCCCCAAGAA<br>GAGCACAGGAGGCΨCΨGGAGGCAGCGGCΨCCGGAGGCΨCΨGGAGGCAGCGGCΨCCGAGAAGGCCGCCAAGG<br>CCGAGGAGGCCGCCAGGAAGAΨGGAGGAGCΨGΨΨCAAGAAGCACAAGAΨCGΨGGCCGΨGCΨGAGGGCCAAC<br>AGCGΨGGAGGAGGCCAΨCGAGAAGGCAGΨGGCCGΨGΨΨCGCAGGAGGAGΨGCACCΨGAΨCGAGAΨCACCΨ<br>ΨΨACAGΨGCCAGACGCCGAΨACCGΨGAΨCAAGGCCCΨGΨCCGΨGCΨGAAGGAGAAGGGAGCAAΨCAΨCGGA<br>GCAGGCACCGΨGACAΨCΨGΨGGAGCAGGCAAGGAAGGCAGΨGGAGΨCΨGGAGCCGAGΨΨΨAΨCGΨGAGCC<br>CCCACCΨGGACGAGGAGAΨCΨCCCAGΨΨCGCCAAGGAGAAGGGCGΨGΨΨΨΨACAΨGCCAGGCGΨGAΨGAC<br>CCCCACAGAGCΨGGΨGAAGGCCAΨGAAGCΨGGGCCACACCAΨCCΨGAAGCΨGΨΨCCCΨGGCGAGGΨGGΨG<br>GGACCCCAGΨΨΨGΨGAAGGCCAΨGAAAGGCCCCΨΨCCCΨAAΨGΨGAAGΨΨΨGΨGCCCACAGGCGGCGΨGA<br>ACCΨGGACAAΨGΨGGCAGAGΨGGΨΨCAAGGCAGGCGΨGCΨGGCCGΨGGGAGΨGGGCAGCGCCCΨGGΨGAA<br>GGGCACCCCΨGAΨGAGGΨGCGGGAGAAGGCCAAGGCCΨΨΨGΨGGAGAAGAΨCAGAGGCGCCACAGAGΨGAΨ<br>GA
**>Membrane-anchored S-2P**<br>AΨGΨΨCGΨGΨΨCCΨGGΨGCΨGCΨGCCΨCΨGGΨGΨCCAGCCAGΨGΨGΨGAACCΨGACCACCAGAACACAGC<br>ΨGCCΨCCAGCCΨACACCAACAGCΨΨΨACCAGAGGCGΨGΨACΨACCCCGACAAGGΨGΨΨCAGAΨCCAGCGΨG

CΨGCACΨCΨACCCAGGACCΨGΨΨCCΨGCCΨΨΨCΨΨCAGCAACGΨGACCΨGGΨΨCCACGCCAΨCCACGΨGΨ
CCGGCACCAAΨGGCACCAAGAGAΨΨCGACAACCCCGΨGCΨGCCCΨΨCAACGACGGGGΨGΨACΨΨΨGCCAGC
ACCGAGAAGΨCCAACAΨCAΨCAGAGGCΨGGAΨCΨΨCGGCACCACACΨGGACAGCAAGACCCAGAGCCΨGCΨG
AΨCGΨGAACAACGCCACCAACGΨGGΨCAΨCAAAGΨGΨGCGAGΨΨCCAGΨΨCΨGCAACGACCCCΨΨCCΨGGG
CGΨCΨACΨACCACAAGAACAACAAGAGCΨGGAΨGGAAAGCGAGΨΨCCGGGΨGΨACAGCAGCGCCAACAACΨG
CACCΨΨCGAGΨACGΨGΨCCCAGCCΨΨΨCCΨGAΨGGACCΨGGAAGGCAAGCAGGGCAACΨΨCAAGAACCΨGC
GCGAGΨΨCGΨGΨΨΨAAGAACAΨCGACGGCΨACΨΨCAAGAΨCΨACAGCAAGCACACCCCΨAΨCAACCΨCGΨG
CGGGAΨCΨGCCΨCAGGGCΨΨCΨCΨGCΨCΨGGAACCCCΨGGΨGGAΨCΨGCCCAΨCGGCAΨCAACAΨCACCC
GGΨΨΨCAGACACΨGCΨGGCCCΨGCACAGAAGCΨACCΨGACACCΨGGCGAΨAGCAGCAGCGGAΨGGACAGCΨ
GGΨGCCGCCGCΨΨACΨAΨGΨGGGCΨACCΨGCAGCCΨAGAACCΨΨCCΨGCΨGAAGΨACAACGAGAACGGCA
CCAΨCACCGACGCCGΨGGAΨΨGΨGCΨCΨGGAΨCCΨCΨGAGCGAGACAAAGΨGCACCCΨGAAGΨCCΨΨCAC
CGΨGGAAAAGGGCAΨCΨACCAGACCAGCAACΨΨCCGGGΨGCAGCCCACCGAAΨCCAΨCGΨGCGGΨΨCCCCA
AΨAΨCACCAAΨCΨGΨGCCCCΨΨCGGCGAGGΨGΨΨCAAΨGCCACCAGAΨΨCGCCΨCΨGΨGΨACGCCΨGGAA
CCGGAAGCGGAΨCAGCAAΨΨGCGΨGGCCGACΨACΨCCGΨGCΨGΨACAACΨCCGCCAGCΨΨCAGCACCΨΨCA
AGΨGCΨACGGCGΨGΨCCCCΨACCAAGCΨGAACGACCΨGΨGCΨΨCACAAACGΨGΨACGCCGACAGCΨΨCGΨ
GAΨCCGGGGAGAΨGAAGΨGCGGCAGAΨΨGCCCCΨGGACAGACAGGCAAGAΨCGCCGACΨACAACΨACAAGC
ΨGCCCGACGACΨΨCACCGGCΨGΨGΨGAΨΨGCCΨGGAACAGCAACAACCΨGGACΨCCAAAGΨCGGCGGCAAC
ΨACAAΨΨACCΨGΨACCGGCΨGΨΨCCGGAAGΨCCAAΨCΨGAAGCCCΨΨCGAGCGGGACAΨCΨCCACCGAGA
ΨCΨAΨCAGGCCGGCAGCACCCCΨΨGΨAACGGCGΨGGAAGGCΨΨCAACΨGCΨACΨΨCCCACΨGCAGΨCCΨA
CGGCΨΨΨCAGCCCACAAAΨGGCGΨGGGCΨAΨCAGCCCΨACAGAGΨGGΨGGΨGCΨGAGCΨΨCGAACΨGCΨG
CAΨGCCCCΨGCCACAGΨGΨGCGGCCCΨAAGAAAAGCACCAAΨCΨCGΨGAAGAACAAAΨGCGΨGAACΨΨCAAC
ΨΨCAACGGCCΨGACCGGCACCGGCGΨGCΨGACAGAGAGCAACAAGAAGΨΨCCΨGCCAΨΨCCAGCAGΨΨΨGG
CCGGGAΨAΨCGCCGAΨACCACAGACGCCGΨΨAGAGAΨCCCCAGACACΨGGAAAΨCCΨGGACAΨCACCCCΨΨ
GCAGCΨΨCGGCGGAGΨGΨCΨGΨGAΨCACCCCΨGGCACCAACACCAGCAAΨCAGGΨGGCAGΨGCΨGΨACCA
GGACGΨGAACΨGΨACCGAAGΨGCCCGΨGGCCAΨΨCACGCCGAΨCAGCΨGACACCΨACAΨGGCGGGΨGΨAC
ΨCCACCGGCAGCAAΨGΨGΨΨΨCAGACCAGAGCCGGCΨGΨCΨGAΨCGGAGCCGAGCACGΨGAACAAΨAGCΨA
CGAGΨGCGACAΨCCCCAΨCGGCGCΨGGAAΨCΨGCGCCAGCΨACCAGACACAGACAAACAGCCCΨCGGAGAGC
CAGAAGCGΨGGCCAGCCAGAGCAΨCAΨΨGCCΨACACAAΨGΨCΨCΨGGGCGCCGAGAACAGCGΨGGCCΨACΨ
CCAACAACΨCΨAΨCGCΨAΨCCCCACCAACΨΨCACCAΨCAGCGΨGACCACAGAGAΨCCΨGCCΨGΨGΨCCAΨG
ACCAAGACCAGCGΨGGACΨGCACCAΨGΨACAΨCΨGCGGCGAΨΨCCACCGAGΨGCΨCCAACCΨGCΨGCΨGCA
GΨACGGCAGCΨΨCΨGCACCCAGCΨGAAΨAGAGCCCΨGACAGGGAΨCGCCGΨGGAACAGGACAAGAACACCCA
AGAGGΨGΨΨCGCCCAAGΨGAAGCAGAΨCΨACAAGACCCCΨCCΨAΨCAAGGACΨΨCGGCGGCΨΨCAAΨΨΨCA
GCCAGAΨΨCΨGCCCGAΨCCΨAGCAAGCCCAGCAAGCGGAGCΨΨCAΨCGAGGACCΨGCΨGΨΨCAACAAAGΨG
ACACΨGGCCGACGCCGGCΨΨCAΨCAAGCAGΨAΨGGCGAΨΨGΨCΨGGGCGACAΨΨGCCGCCAGGGAΨCΨGA
ΨΨΨGCGCCCAGAAGΨΨΨAACGGACΨGACAGΨGCΨGCCΨCCΨCΨGCΨGACCGAΨGAGAΨGAΨCGCCCAGΨA
CACAΨCΨGCCCΨGCΨGGCCGGCACAAΨCACAAGCGGCΨGGACAΨΨΨGGAGCAGGCGCCGCΨCΨGCAGAΨCC
CCΨΨΨGCΨAΨGCAGAΨGGCCΨACCGGΨΨCAACGGCAΨCGGAGΨGACCCAGAAΨGΨGCΨGΨACGAGAACCA
GAAGCΨGAΨCGCCAACCAGΨΨCAACAGCGCCAΨCGGCAAGAΨCCAGGACAGCCΨGAGCAGCACAGCAAGCGC
CCΨGGGAAAGCΨGCAGGACGΨGGΨCAACCAGAAΨGCCCAGGCACΨGAACACCCΨGGΨCAAGCAGCΨGΨCCΨ
CCAACΨΨCGGCGCCAΨCAGCΨCΨGΨGCΨGAACGAΨAΨCCΨGAGCAGACΨGGACCCΨCCΨGAGGCCGAGGΨ
GCAGAΨCGACAGACΨGAΨCACAGGCAGACΨGCAGAGCCΨCCAGACAΨACGΨGACCCAGCAGCΨGAΨCAGAGC
CGCCGAGAΨΨAGAGCCΨCΨGCCAAΨCΨGGCCGCCACCAAGAΨGΨCΨGAGΨGΨGΨGCΨGGGCCAGAGCAAG
AGAGΨGGACΨΨΨΨGCGGCAAGGGCΨACCACCΨGAΨGAGCΨΨCCCΨCAGΨCΨGCCCCΨCACGGCGΨGGΨG
ΨΨΨCΨGCACGΨGACAΨAΨGΨGCCCGCΨCAAGAGAAGAAΨΨΨCACCACCGCΨCCAGCCAΨCΨGCCACGACGG
CAAAGCCCACΨΨΨCCΨAGAGAAGGCGΨGΨΨCGΨGΨCCAACGGCACCCAΨΨGGΨΨCGΨGACACAGCGGAAC
ΨΨCΨACGAGCCCCAGAΨCAΨCACCACCGACAACACCΨΨCGΨGΨCΨGGCAACΨGCGACGΨCGΨGAΨCGGCAΨ
ΨGΨGAACAAΨACCGΨGΨACGACCCΨCΨGCAGCCCGAGCΨGGACAGCΨΨCAAAGAGGAACΨGGACAAGΨACΨ
ΨΨAAGAACCACACAAGCCCCGACGΨGGACCΨGGGCGAΨAΨCAGCGGAAΨCAAΨGCCAGCGΨCGΨGAACAΨC

CAGAAAGAGAΨCGACCGGCΨGAACGAGGΨGGCCAAGAAΨCΨGAACGAGAGCCΨGAΨCGACCΨGCAAGAACΨG
GGGAAGΨACGAGCAGΨACAΨCAAGΨGGCCCΨGGΨACAΨCΨGGCΨGGGCΨΨΨAΨCGCCGGACΨGAΨΨGCCA
ΨCGΨGAΨGGΨCACAAΨCAΨGCΨGΨGΨΨGCAΨGACCAGCΨGCΨGΨAGCΨGCCΨGAAGGGCΨGΨΨGΨAGCΨ
GΨGGCAGCΨGCΨGCAAGΨΨCGACGAGGACGAΨΨCΨGAGCCCGΨGCΨGAAGGGCGΨGAAACΨGCACΨACAC
AΨGAΨGA

>Luciferase
AΨGGAAGAΨGCCAAAAACAΨΨAAGAAGGGCCCAGCGCCAΨΨCΨACCCACΨCGAAGACGGGACCGCCGGCGAG
CAGCΨGCACAAAGCCAΨGAAGCGCΨACGCCCΨGGΨGCCCGGCACCAΨCGCCΨΨΨACCGACGCACAΨAΨCGA
GGΨGGACAΨΨACCΨACGCCGAGΨACΨΨCGAGAΨGAGCGΨΨCGGCΨGGCAGAAGCΨAΨGAAGCGCΨAΨGGG
CΨGAAΨACAAACCAΨCGGAΨCGΨGGΨGΨGCAGCGAGAAΨAGCΨΨGCAGΨΨCΨΨCAΨGCCCGΨGΨΨGGGΨ
GCCCΨGΨΨCAΨCGGΨGΨGGCΨGΨGGCCCCAGCΨAACGACAΨCΨACAACGAGCGCGAGCΨGCΨGAACAGCA
ΨGGGCAΨCAGCCAGCCCACCGΨCGΨAΨΨCGΨGAGCAAGAAAGGGCΨGCAAAAGAΨCCΨCAACGΨGCAAAAG
AAGCΨACCGAΨCAΨACAAAAGAΨCAΨCAΨCAΨGGAΨAGCAAGACCGACΨACCAGGGCΨΨCCAAAGCAΨGΨAC
ACCΨΨCGΨGACΨΨCCCAΨΨΨGCCACCCGGCΨΨCAACGAGΨACGACΨΨCGΨGCCCGAGAGCΨΨCGACCGGG
ACAAAACCAΨCGCCCΨGAΨCAΨGAACAGΨAGΨGGCAGΨACCGGAΨΨGCCCAAGGGCGΨAGCCCΨACCGCAC
CGCACCGCΨΨGΨGΨCCGAΨΨCAGΨCAΨGCCCGCGACCCCAΨCΨΨCGGCAACCAGAΨCAΨCCCCGACACCGC
ΨAΨCCΨCAGCGΨGGΨGCCAΨΨΨCACCACGGCΨΨCGGCAΨGΨΨCACCACGCΨGGGCΨACΨΨGAΨCΨGCGG
CΨΨΨCGGGΨCGΨGCΨCAΨGΨACCGCΨΨCGAGGAGGAGCΨAΨΨCΨΨGCGCAGCΨΨGCAAGACΨAΨAAGAΨ
ΨCAAΨCΨGCCCΨGCΨGGΨGCCCACACΨAΨΨΨAGCΨΨCΨΨCGCΨAAGAGCACΨCΨCAΨCGACAAGΨACGAC
CΨAAGCAACΨΨGCACGAGAΨCGCCAGCGGCGGGGCGCCGCΨCAGCAAGGAGGΨAGGΨGAGGCCGΨGGCCAA
ACGCΨΨCCACCΨACCAGGCAΨCCGACAGGGCΨACGGCCΨGACAGAAACAACCAGCGCCAΨΨCΨGAΨCACCCC
CGAAGGGGACGACAAGCCΨGGCGCAGΨAGGCAAGGΨGGΨGCCCΨΨCΨΨCGAGGCΨAAGGΨGGΨGGACΨΨG
GACACCGGΨAAGACACΨGGGΨGΨGAACCAGCGCGGCGAGCΨGΨGCGΨCCGΨGGCCCCAΨGAΨCAΨGAGCG
GCΨACGΨΨAACAACCCCGAGGCΨACAAACGCΨCΨCAΨCGACAAGGACGGCΨGGCΨGCACAGCGGCGACAΨC
GCCΨACΨGGGACGAGGACGAGCACΨΨCΨΨCAΨCGΨGGACCGGCΨGAAGΨCCCΨGAΨCAAAΨACAAGGGCΨA
CCAGGΨAGCCCCAGCCGAACΨGGAGAGCAΨCCΨGCΨGCAACACCCCAACAΨCΨΨCGACGCCGGGGΨCGCCG
GCCΨGCCCGACGACGAΨGCCGGCGAGCΨGCCCGCCGCAGΨCGΨCGΨGCΨGGAACACGGΨAAAACCAΨGACC
GAGAAGGAGAΨCGΨGGACΨAΨGΨGGCCAGCCAGGΨΨACAACCGCCAAGAAGCΨGCGCGGΨGGΨGΨΨGΨGΨ
ΨCGΨGGACGAGGΨGCCΨAAAGGACΨGACCGGCAAGΨΨGGACGCCCGCAAGAΨCCGCGAGAΨΨCΨCAΨΨAAG
GCCAAGAAGGGCGGCAAGAΨCGCCGΨG

Ψ, N1-methylpseudouridine-5′-triphosphate

Data file S1. Source data for main text figures
Data file S2. Source data for supplemental figures
Data file S3. Statistical analyses
